# Sth1, the Key Subunit of the RSC Chromatin Remodeling Complex, Is Essential in Maintaining Chromosomal Integrity and Mediating High Fidelity Chromosome Segregation in the Human Fungal Pathogen *Candida albicans*

**DOI:** 10.3389/fmicb.2019.01303

**Published:** 2019-06-12

**Authors:** Priya Prasad, Kaustuv Sanyal, Santanu K. Ghosh

**Affiliations:** ^1^Department of Biosciences and Bioengineering, Indian Institute of Technology Bombay, Mumbai, India; ^2^Molecular Mycology Laboratory, Molecular Biology and Genetics Unit, Jawaharlal Nehru Centre for Advanced Scientific Research, Bengaluru, India

**Keywords:** RSC, chromatin remodeling, *Candida albicans*, spindle assembly checkpoint, kinetochore

## Abstract

Chromatin architecture influences gene expression and makes specialized chromatin domains. Factors including histone variants, histone modifiers and chromatin remodelers that define chromatin architecture impact chromosome related processes in *Candida albicans*. In this context, we sought to investigate the roles of the ATP-dependent chromatin remodeler, Remodel the Structure of Chromatin (RSC) in chromosome segregation of *C. albicans*. Sth1 is the key ATPase component of RSC and has profound roles in different cellular processes in *Saccharomyces cerevisiae*. We demonstrate that *STH1* is an essential gene in *C. albicans.* The depletion of Sth1 induces pseudohyphal cells, abnormal spindle morphology, sensitivity toward anti-mitotic drugs and global cohesion defect suggesting an important role of Sth1 in kinetochore-microtubule related processes in *C. albicans*. Strikingly, Sth1 is required to maintain clustered kinetochores revealing the fact that RSC is required in kinetochore integrity. Taken together, we show that RSC plays an important role in various chromatin-templated processes including chromosome segregation in *C. albicans*.

## Introduction

To successfully propagate as a human pathogen, *C. albicans* relies on the events such as to switch between different morphogenic forms, to express myriad virulence-associated genes and to safeguard its DNA from damage made by the host immune system. It has been observed that these events are regulated by epigenetic mechanisms where chromatin architecture plays a pivotal role (Whiteway and Bachewich, 2007). Chromatin organization has been shown to be crucial for faithful chromosome segregation (Sanyal and Carbon, 2002; Sanyal et al., 2004; Baum et al., 2006; Buscaino et al., 2010; Thakur and Sanyal, 2013; Burrack et al., 2016). Several chromatin-associated proteins have emerged as potential targets for generation of new antifungal drugs as resistance to the currently available drugs is on rise (Georgopapadakou, 1998; DiDomenico, 1999; Cowen et al., 2002). In this context, a better understanding of the role of epigenetic factors including histone variants, histone modifiers and chromatin remodelers on the biology of *C. albicans* is needed. The centromeric H3 variant CENP-A^CaCse4^ has been shown to be essential for cell viability and faithful chromosome segregation (Sanyal and Carbon, 2002). Several histone modifying marks and the corresponding writers and erasers of these marks play crucial roles in several biological processes in *C. albicans.* For example, H3K56 acetylation and H3K4 methylation are required for pathogenicity (Lopes da Rosa et al., 2010; Wurtele et al., 2010). Any genetic or pharmacological alteration in the level of acetylated H3K56 reduces the virulence in mice model of *C. albicans* infection (Wurtele et al., 2010). Therefore, the writer and eraser of this mark such as Rtt109 (histone acetyltransferase) and Hst3 (histone deacetylase), respectively were identified as potential drug targets (Wurtele et al., 2010). Similarly, loss of the *SET3C* histone deacetylase complex has been found to cause hyperfilamentation and significantly less mortality during murine systemic candidiasis (Hnisz et al., 2010).

Among the epigenetic factors, the chromatin remodelers provide plasticity to modulate the chromatin architecture. As a result, global gene expression as well as other chromatin templated events such as chromosome segregation, DNA damage repair and replication are regulated by such factors (Clapier and Cairns, 2009). Among the several groups of chromatin remodelers identified in different organisms, SWI/SNF has been shown to be required for the yeast-hyphal transition in *C. albicans* (Mao et al., 2006). Mutants of *SWI1* or *SNF2* are avirulent in the murine systemic candidiasis (Lopes da Rosa and Kaufman, 2012). Since the proteins of this complex in *C. albicans* and humans share significant homology, it is less likely that these proteins may serve as the drug targets. On the other hand, subunits of the RSC complex, the other member of the family of the SWI/SNF chromatin remodeler, share lesser homology to the corresponding proteins of the SWI/SNF complex in human. Thus, unraveling the RSC function in *C. albicans* may lead to the identification of a novel physiological target for the development of anti-fungal drug. The RSC complex is an ATP dependent chromatin remodeler with 17 subunits in *S. cerevisiae* (Cairns et al., 1996; Chambers et al., 2012; Imamura et al., 2015; Sing et al., 2018). Sth1 (**S**NF **T**wo **H**omolog 1) is the catalytic ATPase subunit of RSC (Du et al., 1998) and is homologous to Snf2, the ATPase subunit of the yeast SWI/SNF complex. Unlike SWI/SNF, almost all the proteins of the RSC complex is required for mitotic cell growth (Cairns et al., 1996).

In this study, we describe the functions of the RSC complex in *C. albicans*, for the first time to our knowledge, through functional characterization of Sth1 depletion mutants. We reasoned that the depletion of the catalytic subunit of RSC should inactivate the complex, and hence Sth1 was chosen to study in greater detail. We provide preliminary evidence that Sth1 is indeed a part of chromatin remodeler in *C. albicans* as the *sth1* mutant shows an alteration in the chromatin architecture. Strikingly, the mutant exhibits a defect in centromere clustering that perhaps leads to erroneous kinetochore-microtubule attachment and activation of spindle assembly checkpoint in *C. albicans*. We conclude that RSC through its ability to influence chromatin has significant roles in chromosome segregation in *C. albicans* and is required for the survival of this organism.

## Results

### The ORF C3_02490C Is the Putative *STH1* Gene in *C. albicans* and Is Essential for Cell Survival

**FIGURE 1:**
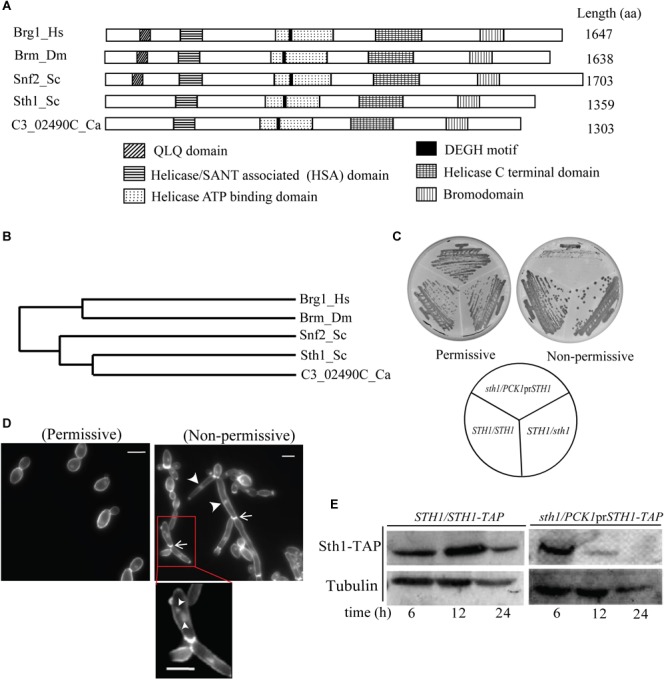
C3_02490C codes for the putative Sth1 in *C. albicans* and is required for cell viability. **(A)** Similarities in the domain organization found in the protein sequence translated from the C3_02490C of *C. albicans*, Sth1 and Snf2 of *S. cerevisiae*, Brm of *D. melanogaster* and Brg1 of *H. sapiens*. The alignment of all the five proteins reveals the bromodomain at the C terminus and a characteristic DEGH motif in the helicase ATP binding domain. **(B)** The phylogenetic tree generated by the Clustal Omega shows that the putative Sth1 of *C. albicans* is most closely related to Sth1 of *S. cerevisiae*, and the two are related to Snf2 of *S. cerevisiae*. These proteins in turn show significant similarities to the proteins in the Snf2-related protein subfamily of other organisms including flies and humans. **(C)** Strain SGC74 (*sth1/PCK1*pr*STH1*) grows well in the permissive (succinate) but fails to grow in the non-permissive (dextrose) medium. SGC6 (*STH1/STH1*) and SGC42 (*sth1/STH1*) grow well in both the media. The plates were incubated at 30°C for 36 h. **(D)** The cell morphology of SGC74 (*sth1/PCK1*pr*STH1*) in the permissive and in the non-permissive media are shown. SGC74 forms budded cells in the permissive medium while it forms elongated and pseudohyphal cells in the non-permissive medium. Arrowheads and arrows show the nuclei and the cell septations, respectively. The inset shows the cells from the non-permissive medium with multiple nuclei marked by arrowheads. Scale bar = 5 μm. **(E)** Immunoblot showing depletion of Sth1. The strain SGC79 (*sth1/PCK1*pr*STH1-TAP*) is grown in the non-permissive medium over a period of 24 h to deplete Sth1. SGC78 (*STH1/STH1-TAP*) is taken as a control which expresses Sth1 grown in permissive as well as non-permissive media.

Based on the amino acid sequence similarity with the Sth1 of *S. cerevisiae*, C3_02490C was identified from the *Candida Genome Database* (CGD^1^) as the putative *STH1* gene in *C. albicans*. The protein translated from C3_02490C was compared to the protein sequences from the members of the SWI/SNF family. Multiple sequence alignment of the protein encoded by C3_02490C of *C. albicans*, Sth1 and Snf2 of *S. cerevisiae*, Brm of *Drosophila melanogaster* and Brg1 of *H. sapiens* revealed a stretch of conserved amino acid sequences which span across the length of the protein (Supplementary Figure S1). Snf2 is the ATPase component of the SWI/SNF chromatin remodeler in yeast, while Brg1 is the ATPase component of the ATP dependent chromatin remodeler BAF complex in humans. Brm is the ATPase component of the Brahma complex in *D. melanogaster*, a multiprotein complex equivalent to the SWI/SNF complex. We found that all these proteins including the protein encoded by C3_02490C harbor the highly conserved domains such as helicase/SANT associated (HSA) domains, helicase ATP binding domain, helicase C terminal domain and bromodomain. We also identified the DEGH motif, a characteristic feature for an ATP binding protein (Linder et al., 1989), in the protein sequence corresponding to C3_02490C and the other proteins to which it was compared (Figure 1A). These domains are important for the functioning of an ATP-dependent chromatin remodeler. Bromodomains are evolutionarily conserved protein-protein interaction modules and serve the fundamental function in recognizing the lysine residues on histones (Fujisawa and Filippakopoulos, 2017) and thus help in targeting of the remodeler to chromatin. The core of the ATP dependent chromatin remodeling enzyme is the helicase-like subunit which harbors domains like SANT associated domain, C-terminal and ATP binding domains. The SANT domain interacts with the histone tails (Boyer et al., 2002; Smith and Peterson, 2005) while the ATP binding domain interacts with the minor groove of AT-rich regions of DNA (Aravind and Landsman, 1998). The DEGH motif is essential for DNA translocase activity (Pereira et al., 2003; Ghosh-Roy et al., 2013). Furthermore, phylogenetic analysis of the protein translated from C3_02490C and the proteins from the Snf2 related protein subfamily from different organisms revealed that Sth1 of *C. albicans* is most closely related to Sth1 of *S. cerevisiae* and both, in turn, are similar to Snf2 of *S. cerevisiae*. Brm of *D. melanogaster* and Brg1 of *H. sapiens* also share significant amino acid sequence similarities with each other, and with the protein translated from C3_02490C, Sth1 and Snf2 of *S. cerevisiae* (Figure 1B). Different putative components of the RSC complex in *C. albicans*, their orthologs in *S. cerevisiae* along with the components of SWI/SNF in *S. cerevisiae* and humans are listed in Supplementary Table S1. These analyses suggest that C3_02490C indeed codes for the ATPase component of RSC which is Sth1 in *C. albicans*.

In *S. cerevisiae, STH1* is an essential gene and cells lacking Sth1 function arrest at the G_2_/M boundary of the cell cycle (Tsuchiya et al., 1998; Hsu et al., 2003). We wished to examine the same for *STH1* in *C. albicans*. To make a mutant of Sth1 in *C. albicans*, we disrupted one of the two alleles of *STH1* using the *SAT1* flipper cassette (Reuss et al., 2004) providing homology of upstream and downstream sequences of *STH1 ORF* for the homologous recombination. The other allele was placed under the control of a regulatable promoter of the *PCK1* gene (Supplementary Figure S2A). The deletion of *STH1* and shuffling of the endogenous promoter with that of *PCK1* were verified by Southern blot hybridization (Supplementary Figure S2B). The resulting strain SGC74 (*sth1/PCK1*pr*STH1*) expresses Sth1 from the *PCK1* promoter which is repressed in presence of glucose and de-repressed when succinate is used as the carbon source. Strains SGC6 (*STH1/STH1*) and SGC42 (*sth1/STH1*) grew similarly on plates containing either succinate or glucose. However, SGC74 (*sth1/PCK1*pr*STH1*) cells grew well on succinate but were unable to grow on glucose medium (Figure 1C), suggesting that like in *S. cerevisiae, STH1* is an essential gene in *C. albicans*. “No growth” phenotype of SGC74 (*sth1/PCK1*pr*STH1*) when the transcription was shut-off was attributed to the absence of the Sth1 protein, as the phenotype was also verified by shuffling the native promoter of *STH1* by the repressible *TET* or *MET3* promoter (Supplementary Figure S3). In all these cases, when the conditional promoter was shut-off, the cells exhibited “no growth” phenotype on agar plates. We further investigated the phenotypes of the cells in Sth1 depleted conditions. In *C. albicans*, cellular stress leads to a change in the cell morphology from yeast to branched and/or elongated forms (Bachewich et al., 2005; Roy and Sanyal, 2011; Thakur and Sanyal, 2011; Mitra et al., 2014). SGC74 (*sth1/PCK1*pr*STH1*) yielded healthy colonies with budded yeast cells when grown in permissive media (YPSU) but under non-permissive conditions (YPDU), SGC74 produced microcolonies. These microcolonies were found to comprise of the cells with filamentous structures and multiple branching phenotype (Figure 1D). We stained the chitin of the cells using calcofluor white (CFW) and found septation along the filaments (Figure 1D). We found constrictions present at the site of each septum that suggests that *sth1* mutant cells produced a pseudohyphal phenotype. Upon nuclear staining using DAPI, we noticed one to multiple nuclei per cell compartment in some cases (shown by arrowheads) (Figure 1D). To confirm that the above phenotype is due to depletion of Sth1 when the conditional knockout strain was grown in non-permissive condition, we examined the expression level of Sth1 in the non-permissive medium where the protein was expressed as a fusion with TAP at the C terminus. Following growth in the non-permissive liquid medium for about 12 h, the protein was grossly depleted in the conditional *sth1* mutant and Sth1 was completely depleted after 24 h of non-permissive growth (Figure 1E). Taken together, these results suggest that the depletion of Sth1 causes a stress-induced elongated pseudohyphal phenotype.

### Sth1 Depletion Causes Cell Cycle Arrest Through Spindle Assembly Checkpoint

**FIGURE 2:**
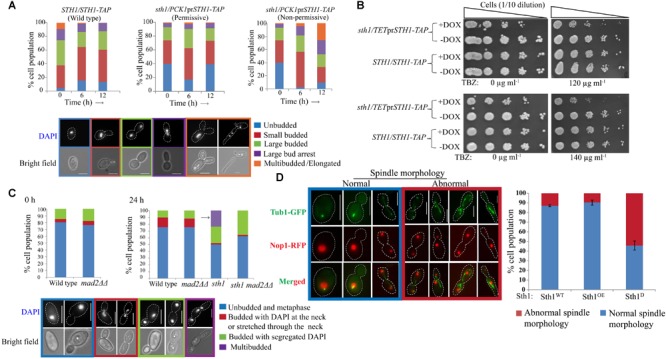
*sth1* mutants activate spindle assembly checkpoint. **(A)** The distribution of cell populations with respect to the bud sizes and nuclei positions are shown for the strains SGC78 (*STH1/STH1-TAP*) and SGC79 (*sth1/PCK1*pr*STH1-TAP*) grown in permissive or non-permissive condition for indicated time intervals. N∼300. Experiments were done in duplicate and the values represent the average from the two experiments. **(B)** Sth1 depleted cells (SGC196, *sth1/TET*pr*STH1-TAP*, grown in presence of doxycycline) show sensitivity toward the microtubule-disrupting agent thiabendazole. Both the strains are grown in permissive (–DOX) and non-permissive (+DOX) medium to partially deplete Sth1 for ∼6 h. 0.5 × 10^7^ cells are washed with water once, 10 fold serially diluted and spotted over the YPDU plates (–DOX) supplemented with TBZ or DMF as mentioned. Plates are incubated at 30°C for ∼36 h and images are taken. **(C)** The deletion of *MAD2* could relieve the stress induced multibudded phenotype (shown by the arrow) in the cells depleted of Sth1 (SGC82, *sth1/PCK1*pr*STH1 mad2ΔΔ* grown in non-permissive medium) for ∼24 h. N∼130. **(D)** Sth1 is required for tubulin morphogenesis. Cells depleted of Sth1 (Sth1^D^; SGC234, *sth1/PCK1*pr*STH1 TUB1-GFP NOP1-RFP* grown in non-permissive medium) show abnormal spindle morphologies in about ∼55% (N∼250) of the cell population. Scale bar = 5 μm.

The requirement of Sth1 for the mitotic cell growth implies that its function is required for continuation of the cell cycle in *C. albicans*. Therefore, we wished to identify the stage(s) of the cell cycle at which this protein may function. We studied the budding index and localization of nuclei in the conditional mutant strain grown in the non-permissive medium. From the population of the asynchronous cells harvested at different time intervals from the non-permissive medium, we found an accumulation of multibudded and elongated pseudohyphal cell population (∼26%, N∼300) when Sth1 was depleted for 12 h (Figure 2A). We observed that the conditional mutant grown in the permissive medium has a higher population (40 and 39%) of unbudded cells than the wild type (4 and 13%) at 0 and 12 h, respectively (Figure 2A). This difference perhaps could arise due to a higher level of expression of Sth1 from the *PCK1* promoter than the physiological endogenous level of the protein from its native promoter. In *C. albicans*, cell cycle perturbations during S or M phase cause the arrest of the cell cycle progression at the S or G_2_/M, respectively leading to a polarized growth of the cell (Bachewich et al., 2005). Therefore, from the increased occurrence of multibudded and elongated pseudohyphal cell phenotypes, we believe that *C. albicans* cells perhaps arrest at the S or G_2_/M phase in Sth1 depleted cells.

Next, we investigated if the arrest is concomitant with the activation of spindle assembly checkpoint (SAC). We reasoned that if *sth1* mutant activates SAC, the mutant would be sensitive to anti-mitotic drugs like thiabendazole (TBZ). Further, the arrest phenotype of the mutant would be ameliorated in combination with a SAC mutation. A conditional mutant of *STH1* under the control of a repressible *TET* promoter that can be turned off using doxycycline (DOX) was used to test the sensitivity of the mutant to TBZ. We observed that the mutant cells showed sensitivity to 120 μg ml^-1^ or higher concentration of TBZ (Figure 2B). The wild type cells, as expected, did not show any sensitivity at the tested concentrations of TBZ. This suggests that the lack of Sth1 function may activate the SAC pathway. If this is true, the arrest phenotype of *sth1* mutant should be relieved in a SAC mutant. To examine this, *MAD2* was deleted in the cells depleted of Sth1 and we found that the removal of Mad2 could relieve stress-induced multibudded and elongated cell phenotypes when Sth1 was depleted for 24 h. However, the same phenotype was found persistent in the population of cells depleted of Sth1 but in presence of Mad2 (Figure 2C). Taken together the facts that the *sth1* mutant is sensitive to TBZ and leads to an activation of SAC suggest that the Sth1 is involved in the kinetochore-microtubule related processes.

A defect in the kinetochore integrity and consequently perturbations in its attachment to microtubules can, in turn, affect the spindle morphology. Therefore, we investigated the spindle morphology in the cells depleted of Sth1 where tubulin (Tub1) was fused with GFP and as a nuclear marker, nucleolar protein Nop1 was fused with RFP. We found a considerable increase in the population of the *sth1* mutant cells with abnormal spindle morphologies such as elongated, short/undivided and mis-oriented spindles along with abundant cytoplasmic microtubules as compared to the wild type cells (Figure 2D, red box). The normal spindle morphologies were visible as the dot-like signals in the unbudded cells, short and long rod-like signals in the metaphase and the anaphase cells, respectively (Figure 2D, blue box). From these results, we conclude that the perturbation in kinetochore and spindle functions may be the reasons to cause SAC-dependent cell cycle arrest in the depleted levels of Sth1 in *C. albicans*.

### Sth1 Has Chromatin Remodeling Function in *C. albicans*

**FIGURE 3:**
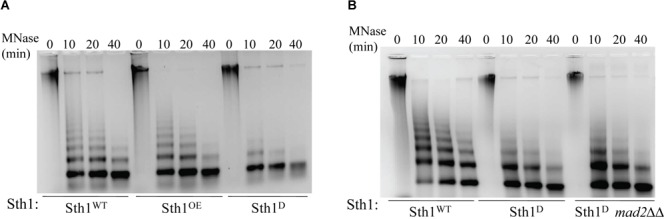
Chromatin architecture in wild type and in cells with altered level of Sth1. **(A)** Agarose gel stained with ethidium bromide showing MNase digestion of the global chromatin from the wild type (Sth1^WT^) and the cells overexpressing Sth1 (Sth1^OE^) or depleted of Sth1 (Sth1^D^). Unlike wild type, lack of long nucleosome ladder is observed in the cells depleted for Sth1. **(B)** MNase digested chromatin of Sth1 depleted cells in presence or absence of Mad2 show a similar profile, where the long ladder of nucleosomes is missing.

If Sth1 indeed is a component of the chromatin remodeler, it should modulate the chromatin architecture in *C. albicans*. To examine this, we analyzed the global chromatin structure in the cells in presence or absence of Sth1 by digesting chromatin using micrococcal nuclease (MNase) (Keene, 1981). Equal units of MNase (0.375 U) were added per reaction which was incubated at 37°C for the increasing period (0, 10, 20, and 40 min). This was followed by deproteination of chromatin and analyzing the purified DNA on an agarose gel by staining with ethidium bromide (EtBr) which revealed that the global chromatin structure is altered when Sth1 is depleted as compared to the wild type cells or cells overexpressing Sth1. While in the latter two cases, distinct long DNA ladders corresponding up to 8 to 9 nucleosomes were visible (Figure 3A, wild type and overexpression of Sth1; 10 and 20 min of digestion by MNase), bands corresponding to mostly mono- and some di-nucleosomes were only visible in the cells depleted of Sth1 (Figure 3A, depletion of Sth1; 10, 20, and 40 min of digestion by MNase) suggesting a gross alteration in the global chromatin structure. This result indicates that Sth1 has the ability to remodel the chromatin architecture in *C. albicans*. Further, to verify that the altered chromatin structure observed in *sth1* is not due to an elongated and multibudded morphology of the cells that occurs when Sth1 is depleted, we examined the global chromatin structure in a similar way in *mad2ΔΔ* cells depleted of Sth1, where the stress-induced multibudded and elongated phenotype was not found (Figure 2C). We observed that unlike wild type, chromatin of Sth1 depleted cells in presence or absence of Mad2 showed similar patterns when digested by MNase (Figure 3B). This suggests that it is the lack of Sth1 function but not cellular morphology *per se* that leads to the alteration in the chromatin architecture. With an ability to remodel the global chromatin architecture, depletion of Sth1 is expected to have pleiotropic effects. In the subsequent studies, we focused our investigation on the role of RSC in chromosome segregation.

### Sth1 Is Enriched at Centromeric Chromatin and Is Required for Kinetochore Clustering

**FIGURE 4:**
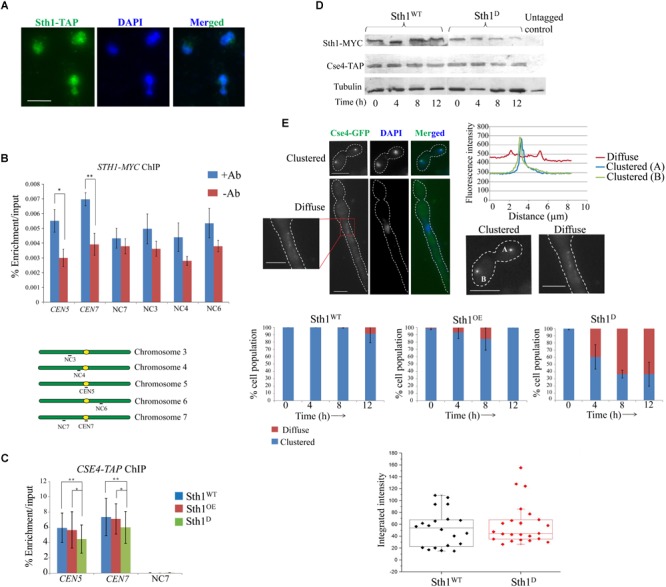
Sth1 is a nuclear protein binding to the centromeric chromatin and is required for the clustering of centromeres. **(A)** Sth1 completely localizes within the nucleus throughout the cell cycle. The wild type strain SGC78 (*STH1/STH1-TAP*) is grown till the log phase, fixed with 5% formaldehyde and processed for immunostaining (as described in materials and methods). Scale bar = 5 μm. **(B)** Chromatin immunoprecipitation (ChIP) is performed with the strains SGC129 (*STH1/STH1-MYC*) using anti-c-myc antibodies (as described in experimental procedures). Relative enrichments of Sth1 at the *CEN5, CEN7* and non-centromeric regions on different chromosome are measured. Sequences of the primer pairs used to amplify these regions are mentioned in Supplementary Table S3. *P*-values for the two-tailed paired *t*-test to show the statistical significance between the two compared values are as follows: ^∗^*p* = 0.005, ^∗∗^*p* = 0.003. **(C)** Relative enrichment of Cse4-TAP at the centromeric loci does not vary with changes in the level of Sth1 [depletion or overexpression, (SGC262, *sth1/PCK1*pr*STH1-MYC CSE4-TAP* grown in non-permissive and permissive media, respectively)]. PCR is performed from the immunoprecipitated DNA using primer pairs from the *CEN5, CEN7* and the non-centromeric region (NC7). *P*-values for the two-tailed paired *t*-test to show the statistical significance between the two compared values are as follow: ^∗^*p* = 0.364, ^∗∗^*p* = 0.794. **(D)** Expression of Cse4-TAP does not vary with changes in the level of Sth1. **(E)** The top left panel shows signal of Cse4-GFP analyzed to reveal its subcellular localization in the wild type and in the *sth1* mutant. Clustered or diffuse localization of Cse4-GFP is observed as shown. Scale bar = 5 μm. Top right panel shows the fluorescence intensity of the individual clustered or the diffuse signal. Middle panel shows that with increasing time of depletion of Sth1, the population of *sth1* cells with the diffuse signal of Cse4-GFP increases and after 12 h of depletion of Sth1, ∼60% (N∼110) of the cells show diffuse signal. Bottom panel shows that the integrated intensity of Cse4-GFP does not vary between the wild type (SGC260, *STH1*/*STH1-MYC CSE4/CSE4-TAP*) and the *sth1* mutant cells (SGC262, *sth1/PCK1*pr*STH1-MYC CSE4-TAP* grown in non-permissive medium). Integrated intensity of Cse4-GFP is calculated using ImageJ software by subtracting the background intensity from the Cse4-GFP intensity and the normalized values are plotted in the box plot.

In accord to its probable functions on kinetochore-microtubule related processes, it is expected that Sth1 (RSC) would act as a chromatin remodeler and would reside within the nucleus in *C. albicans*. Consistent with this, we observed Sth1 within the nucleus throughout the cell cycle (Figure 4A). Further chromatin immunoprecipitation (ChIP) assays revealed that Sth1 binds to the centromere, although its binding to the non-centromeric chromosomal arm regions could not be detected (Figure 4B). However, it is possible that the binding of Sth1 at the arm regions is too weak to be detected by our assay. These weak interactions could be due to either inefficient cross-linking or transient presence of RSC at these regions during the remodeling activities as shown for several other chromatin remodelers including RSC in *S. cerevisiae* (Ng et al., 2002; Whitehouse et al., 2007; Yen et al., 2012; Kubik et al., 2018).

Sensitivity toward anti-microtubule drug, spindle assembly checkpoint-dependent cell cycle arrest and occupancy of Sth1 at centromeres suggest that RSC might have a role at the centromere/kinetochore complex in *C. albicans*. Sth1 has been found to be present at the centromere and the pericentromere regions and interact with kinetochore components in *S. cerevisiae* (Hsu et al., 2003) implicating that RSC is required for proper kinetochore function and faithful chromosome segregation. To investigate the role of Sth1 on kinetochore function in *C. albicans* we analyzed centromere localization of CENP-A (CaCse4), as a marker to measure the kinetochore integrity (Thakur and Sanyal, 2012) in the cells lacking Sth1. We failed to detect any mis-targeting of CENP-A when Sth1 was either depleted or overexpressed (Figure 4C). Consistent with this, the level of expression of CENP-A was also found to be indistinguishable in Sth1 depleted cells when compared to the wild type (Figure 4D). Strikingly, when we compared the signals of CENP-A (Cse4-GFP) in the wild type and the *sth1* mutant, the signals appeared, either clustered or diffuse as shown in Figure 4E, (top left panel). To compare the signal intensities, signals appearing from one each of clustered or diffuse kinetochores were plotted that depicts a decrease in the signal intensity of the diffuse type compared to the clustered type (Figure 4E, top right panel). We observed in about 60% (N∼110) of the mutant cells, signals of CENP-A (Cse4-GFP) are diffuse as opposed to a bright, tight-knit dot-like signal representing a kinetochore cluster in 90% (N∼110) of the wild type cells (Figure 4E, middle panel). However, we found that the integrated intensity of CENP-A (Cse4-GFP), when quantified using ImageJ software, remains the same in the wild type and the *sth1* mutant cells (Figure 4E, bottom panel) suggesting the levels of total or targeted CENP-A (Cse4-GFP) do not vary between the wild type and the mutant as observed before (Figure 4C,D). These results indicate that in the absence of Sth1, the kinetochores in *C. albicans* may be declustered causing a diffuse Cse4-GFP signal whereas localization of the protein to individual centromeres remains intact. The diffuse CENP-A (Cse4-GFP) is generated due to the depletion of Sth1 and not due to the altered cellular morphology of the *sth1* mutant as a tight-knit clustered signal of CENP-A (Cse4-GFP) was observed in the wild type cells induced to form filamentous hyphae phenotype (Supplementary Figure S4). Nevertheless, such a centromere declustering associated with the Sth1 depleted cells can potentially cause a defect in kinetochore-microtubule interactions and may lead to mis-segregation of the chromosomes.

### Mcd1 Association to the Centromeric Region Decreases in *RSC* Mutants

**FIGURE 5:**
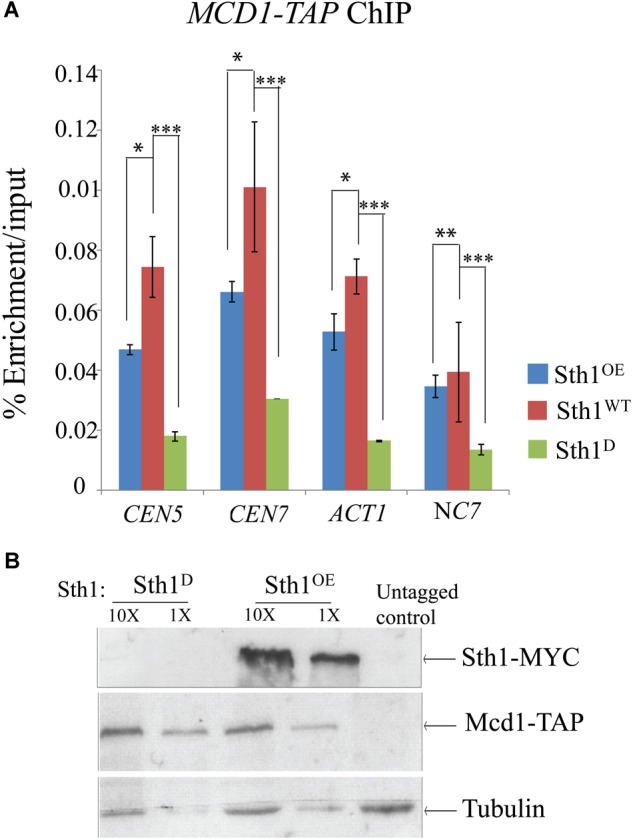
Mcd1 binding to centromeric and non-centromeric regions decreases with alteration in the Sth1 level. **(A)** ChIP experiment showing decreased relative enrichment of Mcd1-TAP at the centromeric and non-centromeric loci in the cells with an altered level of expression of Sth1. *P*-values for the two-tailed paired *t*-test to show the statistical significance between the two compared values are as follows: ^∗^*p* < 0.05, ^∗∗^*p* > 0.05, ^∗∗∗^*p* < 0.05. **(B)** Immunoblot showing the Mcd1 level remains unchanged in the cells with depletion [SGC120 (*sth1/PCK1*pr*STH1-MYC MCD1/MCD1- TAP*) grown in non-permissive medium] or overexpression (SGC120 grown in permissive medium) of Sth1.

Earlier studies in *S. cerevisiae* have demonstrated that cohesins fail to associate with centromeres and with chromosomal arms causing a defect in sister chromatid cohesion in the *sth1* mutant (Huang et al., 2004; Lopez-Serra et al., 2014). Since the centromeres of *S. cerevisiae* and *C. albicans* differ in size, organization as well as the structure of centromeric chromatin (Fitzgerald-Hayes et al., 1982; Hieter et al., 1985; Steiner and Clarke, 1994; Baum et al., 2006) and the fact that centromere clustering is compromised in the *sth1* mutant in *C. albicans* (Figure 4E), we next investigated how the lack of RSC might affect the cohesin association with the epigenetically determined centromeres in *C. albicans*. Using ChIP assays, we indeed observed a considerable reduction in the association of Mcd1 both at the centromere and the non-centromeric arm regions in the cells depleted of Sth1 as compared to the wild type cells (Figure 5A). Notably, the Mcd1 level at the centromere was also found reduced in the cells overexpressing Sth1 (Figure 5A). However, such a reduction was not observed in all the non-centromeric regions in the cells overexpressing Sth1. For example, a reduction was observed at the *ACT1* locus but not at the intergenic NC7 locus. Since an improper chromatin remodeling function can alter gene expression, we wanted to test if the reduced binding of Mcd1 at the centromere is due to a global reduction in the Mcd1 expression in the cells where Sth1 is depleted or overexpressed. However, we observed that the Mcd1 level remains unchanged under both these conditions (Figure 5B). From these results, we conclude that maintenance of homeostasis of RSC-mediated chromatin remodeling activity both at the centromeres and the arm regions may be crucial for the recruitment of cohesin in *C. albicans*.

## Discussion

In this study, we characterized the effect of depletion of Sth1, the ATPase of the RSC chromatin remodeling complex in *C. albicans* identified by *in silico* analysis from CGD. Besides *in silico* analysis, a separate study has identified different proteins, co-purified with CaSth1, and are annotated in the CGD as putative components of the RSC complex (will be described elsewhere), suggesting that CaSth1 is indeed the part of the RSC complex in *C. albicans*. Using three repressible promoters (*PCK1, MET3, TET*), we convincingly demonstrated that Sth1 is essential for the survival of *C. albicans*. The alteration of the global chromatin structure in cells depleted of Sth1 indicates its chromatin remodeler function in *C. albicans*. Given important roles Sth1 plays in cell cycle progression and chromosome segregation in *S. cerevisiae* (Tsuchiya et al., 1998; Hsu et al., 2003), we speculated that Sth1 in *C. albicans* would have a significant role to play during the cell cycle. Indeed, depletion of this protein transiently arrests the cells at the G_2_/M stage through activation of spindle assembly checkpoint as demonstrated in *S. cerevisiae* (Tsuchiya et al., 1998; Hsu et al., 2003). Prolonged depletion of Sth1 resulted in multibudded and elongated cells suggestive of S or G_2_/M arrest which became largely ameliorated upon removal of the SAC protein, Mad2. Notably, we failed to observe a high percentage of S or G_2_/M arrested cells in *C. albicans* in contrast to the observation in *S. cerevisiae* (Tsuchiya et al., 1998; Hsu et al., 2003). A probable reason would be as we depleted Sth1 in an asynchronous population of the cells, they were arrested at different stages of the cell cycle whereas in *S. cerevisiae* with a similar population of the cells, the cell cycle arrest was observed only at the G_2_/M boundary in the *sth1* mutant (Hsu et al., 2003). This assumes that RSC might have broader roles across the stages of the cell cycle in *C. albicans* than in *S. cerevisiae*. With a global remodeling activity, RSC proteins have been shown to bind different regions across the genome (Floer et al., 2010; Wippo et al., 2011; Ganguli et al., 2014) including at the centromeres in a cell cycle dependent manner as well as at the partitioning locus of the endogenous 2 micron plasmid in *S. cerevisiae* (Huang et al., 2004; Ma et al., 2013). Although we observed an association of RSC with the centromeres, no such association was detected with the chromosomal arm regions. Nevertheless, from these results, the functions of RSC in kinetochore-microtubule related processes appear to be conserved across *S. cerevisiae* and *C. albicans* despite their centromeres vary in size and organization. Strikingly, unlike in *S. cerevisiae*, we observed a compromised kinetochore clustering in the *sth1* mutant in *C. albicans*. In contrast to the previous reports that any declustering of the centromeres in *C. albicans*, primarily due to loss of outer or middle kinetochore proteins, can destabilize Cse4 from the centromere (Thakur and Sanyal, 2011, 2012), we failed to observe any destabilization of Cse4 in the *sth1* mutant. This result suggests while Sth1 is required for kinetochore clustering but not kinetochore stability. Similarly, no CENP-A (ScCse4) mislocalization has been observed in the *sth1* mutant in *S. cerevisiae*. Since lack of Sth1 in *C. albicans* causes a defect in the global chromatin architecture, regional centromeres in *C. albicans* presumably containing both canonical and CENP-A nucleosomes may be perturbed leading to centromere declustering.

An alteration in the chromatin environment can affect other chromatin driven events such as stabilization of spindle microtubules (Sampath et al., 2004) and sister chromatid cohesion (Tanaka et al., 1999; Partridge et al., 2001; Kim et al., 2002; Nonaka et al., 2002; Unal et al., 2004; Chang et al., 2005; Onn et al., 2008). In accord to this, we observed a gross spindle abnormality such as short and misaligned spindles in the *sth1* mutant. The abnormal spindle morphology was also observed in the *sth1* mutant in *S. cerevisiae* (Hsu et al., 2003). Further, we found a defect in cohesin-chromatin association (Hsu et al., 2003) as reported in *S. cerevisiae* through negating RSC-dependent cohesion loader (Scc4, Scc4) recruitment (Lopez-Serra et al., 2014). At this moment we cannot distinguish whether the *sth1* depletion directly or through the cohesin loader causes the reduction in cohesin recruitment in *C. albicans*. We also cannot exclude the possibility that the observed effect of Sth1 on cohesin or at the centromeres may be an indirect effect of alteration in transcription of the genes involved in these processes as RSC is known to regulate transcription. In this regard, we could not detect any change in expression of at least Mcd1 (cohesin) or Cse4 in the *sth1* mutant. However, in *S. cerevisiae*, several of the mitotic genes were found to be unaffected by RSC inactivation (Cao et al., 1997) suggesting this complex might have some direct functions during the cell cycle. Additionally, proteins of chromatin remodelers have also been observed at the centrosomes regulating recruitment of centrosomal proteins and microtubule nucleation that appears to be transcription independent events (Sillibourne et al., 2007). Regardless of the mechanism involved, it is clear that similar to *S. cerevisiae*, the loss of RSC function can cause centromere and cohesion defects leading to chromosome missegregation in *C. albicans*.

Throughout this study, to assess the function of Sth1, we analyzed the cell cycle by depleting it by the repressible promoters. We relied on these widely tested methods of depletion of protein in *C. albicans* instead of recently developed “anchor away” technique adopted in *S. cerevisiae* for fast inactivation of RSC proteins (Kubik et al., 2018) due to the non-availability of this technique optimized for *C. albicans*. Development of such a technique for *C. albicans* is beyond the scope of this report. From our results, it can be envisaged that RSC function is required for proper chromatin architecture that helps in chromatin driven events including centromere function and cohesin recruitment. For these processes, RSC is believed to maintain a dynamic chromatin state by moving, ejecting or restructuring the nucleosomes using the energy of ATP hydrolysis. While our data suggest that Sth1 influences the global chromatin architecture, we could not detect any significant Sth1 interaction at the non-centromeric loci perhaps due to the transient catalytic activity of the remodeler at these loci. It has been recognized that assaying chromatin binding by chromatin remodelers by ChIP is challenging due to inefficient cross-linking and transient nature of their association with chromatin. In spite of this, several subunits of different remodelers including Snf2, a close homolog of Sth1, have been successfully assayed as they are believed to remain close to the DNA (Yen et al., 2012). In absence of any information regarding the composition of the RSC subunits and their relative proximity to the DNA in *C. albicans*, we relied on ChIP assay for Sth1 localization assuming this also remains close to DNA like its homolog (Snf2) in *S. cerevisiae*. We indeed observed Sth1 at the centromeres by ChIP assay. Notably, we do believe that Sth1 in *C. albicans* can also bind to the gene promoters (Ng et al., 2002; Ramachandran et al., 2015) and the coding regions of the highly transcribed genes (Spain et al., 2014) as observed in *S. cerevisiae*. However, in this report, as we focus on revealing the role of RSC in chromosome segregation in *C. albicans*, studying the transcription-related functions of this complex is yet another dimension to explore further.

Overall, this study uncovers that the RSC complex is an epigenetic determinant for proper chromatin architecture in *C. albicans* and thus has significant importance in chromosome segregation. Comparing our results with that available from *S. cerevisiae*, we speculate that the RSC complex might have rewired in *C. albicans* in a way to accommodate additional functions, particularly at the centromeres perhaps because these loci vary epigenetically between these two fungi. However, the functions of the RSC complex on overall chromosome segregation appear to be similar in both these fungi. Future studies leading to the characterization of other subunits of RSC complex, identification of the RSC target loci across the genome and analysis of the transcriptome of the mutant followed by a comparison between these two data sets would reveal the spectrum of functions of this complex in other aspects of biology of *C. albicans* including DNA damage repair, morphogenesis, commensalism, stress response and pathogenicity. Importantly, the component proteins of RSC share lesser homology with the human host proteins compared to the well studied SWI/SNF chromatin remodeling complex proteins.

Due to increasing resistance to the currently available anti-Candida drugs, demands for the novel anti-fungal drug are high. To meet this, studies to search for any alternative physiological target that can affect the survival and pathogenic fitness of this organism are of great importance. For the last several years with the revelation of chromatin-regulated virulence factors in fungal pathogenesis, chromatin proteins have been identified as promising antifungal targets (Rai et al., 2018). In this context, we sought to characterize the RSC chromatin remodeling complex to reveal its cell cycle related functions for the first time in the biology of *C. albicans*. Given an increasing occurrence of resistance against anti-Candida drugs, the subunits of the RSC complex might provide novel targets to develop more potent and safer anti-fungal drugs. Though there is a high degree of sequence similarity between Sth1 and its homolog protein Brg1 in humans, there are several other components of RSC complex which do not have homologs in humans and hence can be potential drug targets. Among these, *RSC4* was found to be a non-essential gene with a crucial role in morphogenesis in *C. albicans* (unpublished data). Similarly, *RSC9* was found to be an essential gene having a crucial role in nuclear segregation in *C. albicans* (unpublished data). The orthologs of these genes in the related fungi *S. cerevisiae* have important functions in cell wall integrity (Kasten et al., 2004) and oxidative stress response (Damelin et al., 2002) which are clinically significant for *C. albicans* infection.

## Materials and Methods

### Strains, Media, Growth Conditions and Plasmids

*Candida albicans* strains used in this study are listed in Supplementary Table S2. *C. albicans* cells were grown in YPD (1% Yeast extract, 2% peptone, and 2% dextrose) supplemented with 0.01% uridine or SCD (synthetic complete dextrose with 0.67% YNB and 2% dextrose). 2% succinate was used as carbon source to overexpress the protein using *PCK1* promoter. The primers used in this study are listed in Supplementary Table S3. Plasmids used in this study are listed in Supplementary Table S4. All *C. albicans* strains were grown at 30°C unless otherwise stated.

### Transformation of *C. albicans*

*Candida albicans* transformation was performed by the LiAc method mentioned elsewhere (Wilson et al., 1999). Briefly, strains to be transformed were inoculated into YPDU or YPSU and grown overnight (12–16 h) at 30°C. The pre-culture was diluted into fresh respective YPDU (or YPSU) medium such that initial OD_600_ of the culture is ∼0.2 and subsequently the cells were grown for 3–4 h at 30°C. When OD_600_ finally reaches ∼1, cells were harvested and washed with water followed by TE-LiAc (10 mM Tris pH 7.5, 1 mM EDTA, 100 mM LiAc). 50 μg sheared salmon sperm DNA was added to each system followed by the DNA to be introduced. About 3 volumes of PEG mix (42% PEG, 10 mM Tris pH 7.5, 1 mM EDTA, 100 mM LiAc) were then added and the transformation mix was incubated for 14–16 h at 30°C. Transformation mix was given heat shock at 42°C for 45 min; PEG was washed off and recovered in YPDU/YPSU for 1 h (except *NAT1* marker transformations that were recovered for 3 to 4 h). Finally, cells were plated onto the selective media plates and incubated for about 48 h. Transformants colonies were screened to confirm the correct integration.

### Chromatin Immunoprecipitation Assay and qPCR

This was performed as described before (Braunstein et al., 1993). Typically, 5 × 10^8^ cells were harvested and fixed by formaldehyde for 15 to 60 min (depending on the protein being cross-linked). Growth regime followed for depleting the protein (Sth1) for the ChIP experiment is as follows. The cells were freshly diluted in the non-permissive medium (YPDU) from the overnight grown culture. The partial depletion of the protein (Sth1) in the non-permissive medium was done for 12 to 14 h, and the culture was again repassaged in the fresh non-permissive medium setting the initial OD_600_ as 0.2–0.3, allowing it to grow for 4–5 h such that final OD_600_ reaches ∼1. The wild type cells were grown following a similar regime. Finally, the cells were harvested and proceeded for the experiment. Spheroplasts were made from the fixed cells followed by sonication with 21 s ON and 1 min OFF on ice for 13 rounds to shear the chromatin yielding fragmented DNA in the range of 300–500 bp. Anti-protein A (Sigma, Cat. No. P3775) or anti-c-myc (Rabbit polyclonal, Abcam, United Kingdom) antibodies were used at a final concentration of 5 μg ml^-1^. Protein-DNA were de-crosslinked at 65°C for 14–16 h and DNA was purified using SureExtract PCR purification kit (Nucleo-pore, Genetix, India) as per the manufacturer’s instructions and eluted in 50 μl of elution buffer. qPCR was performed on the Bio-Rad CFX96 Real Time System with iTaq Universal Sybr Green Supermix (Bio-Rad, United States). Calculations for Enrichment/Input values were made as described elsewhere (Fernius and Marston, 2009) using the given equation: ΔC_t_ = C_t_(ChIP)-{C_t_(Input) – logE (Input dilution factor)}, where E is the value of specific primer efficiency; Enrichment/Input = E^∧-ΔCt^. Error bars were calculated from the standard deviation from two technical replicates from at least two independently grown cultures. Information about the primers and their relevant sequences are given in Supplementary Table S3.

### Making of Spheroplast

To make spheroplast, 5 × 10^8^ wild type cells were harvested and washed once with spheroplasting buffer (20 mM Na-HEPES pH 7.4, 1.2 M Sorbitol) and were finally resuspended in 10 ml of spheroplasting buffer. 2.5 μM of β-mercaptoethanol and 50 μg ml^-1^ of 20T Zymolyase were added to the cell suspension which was incubated at 30°C in shaking condition of 65 rpm for about 45 min. Spheroplast formation was checked by taking the OD_600_ in 5% SDS before and after 45 min of incubation with Zymolyase. A drop of more than 95% in OD_600_ suggests that spheroplast formation has happened in almost all the cells and was proceeded for the experiments (ChIP, MNase, etc.). We observed that the *sth1* mutant was difficult to make spheroplast using the protocol mentioned above. To make spheroplast from *sth1*, we added twice the amount of Zymolyase (100 μg ml^-1^) and the incubation time was extended to 1–1.5 h. However, the wild type cells could easily form spheroplasts in 30–45 min.

### Chromatin Digestion by Micrococcal Nuclease (MNase)

Micrococcal nuclease assay was performed as described elsewhere (Marschall and Clarke, 1995). Typically, 4 × 10^9^ cells were harvested and treated with 20T Zymolyase (MP Biomedicals) to make spheroplast. The spheroplasts were resuspended in MNase digestion buffer and were treated with equal units (0.375 units) of MNase enzyme (Sigma, Cat. No. 3755). Digestion was carried out at 37°C for 0, 10, 20, and 40 min. 0.25 mM EDTA supplemented with 5% SDS was used to stop the digestion reaction. DNA was extracted, purified and quantified. 3 μg of purified DNA was electrophoresed on 1.4% agarose gel and was stained using ethidium bromide (EtBr). For the experiment in this study, Sth1 was depleted by growing the strain (*sth1/PCK1*pr*STH1-TAP*) for 12 h in non-permissive medium (YPDU). Similar growth regime was followed for the wild type strain *(STH1/STH1-TAP)* grown in YPDU and YPSU media.

Notably, the number of cells taken for chromatin isolation was different for the wild type and the *sth1* mutant, because the elongated pseudohyphal cells obtained from the mutant were difficult to form spheroplast. Hence to obtain an equal amount of chromatin to start the MNase digestion reaction, twice the volume of cell culture of the mutant than the wild type (both grown for 12 h) was taken. 4 × 10^9^ wild type cells were taken for the isolation of chromatin in the experiment. Hence to begin the MNase digestion reaction, equal amount of chromatin was taken per reaction, as evident by the presence of equal amount of purified undigested DNA at 0 min time point for each sample (Figure 3).

### Protein Extraction and Immunoblot

Cells grown for overnight were freshly diluted into YPDU (or YPSU) to set the initial OD_600_ as 0.2. After growing for required time, cells were harvested and washed with water once to remove the medium. Pellets were boiled for 5 min and were resuspended in ice-cold RIPA buffer (50 mM Tris pH 8, 150 mM NaCl, 1% NP-40, 3 mM EDTA, 0.5% deoxycholate, 0.1% SDS, 10 mM DTT) containing the protease inhibitor cocktail (Sigma) and were lysed with acid-washed glass beads (Sigma) by vigorous vortexing for 1 min followed by keeping the pellet on ice for 1 min, the cycle repeated for 4 times. Lysates were cleared twice by centrifugations at 4°C to remove cell debris and subjected to electrophoresis in 8–12% SDS PAGE. Gels were transferred to a nitrocellulose membrane (Pall life sciences, United States) and blocked in 5% skimmed milk in TBS-T. Membranes were incubated with primary antibodies like anti-Protein A (Sigma, Cat. No. P3775), anti-Myc (9E10, Roche) or anti-tubulin (YOL1/34, Abcam, Cat. No. ab64332) with the recommended concentration in 5% non-fat milk TBS-T. Membranes were washed 3 times in TBS-T and then exposed to the recommended concentration of secondary antibodies (anti-mouse or anti-rabbit horseradish peroxidase antibodies (Pierce, United States) in 5% non-fat milk in TBS-T. Membranes were washed 3 times in TBS-T, and finally once with water. TMB/H2O2 substrate (GeNei, India) at 1:50 dilution in water was added to the blot and allowed for the bands to get developed before capturing the image.

### 4′,6-Diamidino-2-Phenylindole (DAPI) and Calcofluor White (CFW) Staining

Cells were harvested and washed twice with 0.1 M phosphate buffer. They were subsequently fixed with 50% ethanol and again washed twice with 0.1 M phosphate buffer. Finally, the cells were resuspended in 10 μg ml^-1^ DAPI and incubated at 4°C in dark for 10 min.

To the cells washed with 0.1 M phosphate buffer, 5% of 1:1000 diluted CFW was added and the cells were incubated in dark for 10 min at room temperature.

### Subcellular Immunostaining

Log phase cells were harvested and fixed by 5% formaldehyde for 30 min at room temperature. Fixed cells were washed once with 0.1 M phosphate buffer followed by once with spheroplasting solution (1.2 M sorbitol, 0.1 M phosphate buffer pH 7.5) and finally resuspended in the spheroplasting solution. Spheroplast was made using 20T Zymolyase in the presence of 25 mM β-mercaptoethanol for 1 h at 30°C. The spheroplast was washed with spheroplasting solution, transferred to poly-L-lysine coated slides bearing Teflon coated wells. The adhered spheroplasts were flattened and permeabilized by immersing the slide in ice cold methanol followed by ice cold acetone for 5 and 30 s, respectively. Blocking buffer (10 mg ml^-1^ BSA with 5% skim milk prepared in PBS) was added onto each well, incubated for 30 min, followed by 3–4 times washing by PBS. All the primary and secondary antibody dilutions were prepared into antibody dilution buffer (10 mg ml^-1^ BSA in PBS). Spheroplasts were incubated with 1/1000 dilution of primary antibodies (anti-Protein A, Sigma, Cat. No. 3775) for 1 h, followed by washing with PBS for 3–4 times, incubated with 1:500 dilution of secondary antibodies Alexa Fluor 568 Goat anti-rabbit IgG (Invitrogen, United States). After subsequent wash with PBS, DAPI (1 μg ml^-1^ in 0.1 M phosphate buffer) was added to the spheroplast, incubated in dark for 15 min, again washed with PBS and mounted using 90% glycerol supplemented with 1 mg ml^-1^ p-phenylenediamine.

## Author Contributions

PP and SG contributed in conception and design of the study. PP performed the experiments and drafted the manuscript. PP, SG, and KS interpreted and analyzed the results, and critically revised the manuscript.

## Conflict of Interest Statement

The authors declare that the research was conducted in the absence of any commercial or financial relationships that could be construed as a potential conflict of interest.

## Footnotes

1 http://www.candidagenome.org
