# Supplementary material for: Sth1, the Key Subunit of the RSC Chromatin Remodeling Complex, Is Essential in Maintaining Chromosomal Integrity and Mediating High Fidelity Chromosome Segregation in the Human Fungal Pathogen Candida albicans
Source: Front Microbiol. 2019 Jun 12;10:1303. doi: 10.3389/fmicb.2019.01303 (PMC6582774; doi:10.3389/fmicb.2019.01303)
Supplement: Supplementary file 1 [file Data_Sheet_1.doc]

Supplementary Material

1. **Supplementary figures and tables**
   1. **Supplementary figures:** **S1**. C3_02490C of *C. albicans* codes for the putative Sth1. **S2**. Construction of conditional mutant of *sth1* where one of the allele of *STH1* is deleted and the other one is placed under the control of *PCK1* promoter. **S3**. *STH1* is an essential gene in *C. albicans* shown by repressible *MET3* or *TET* promoter. **S4.** Cse4-GFP remains clustered in the filamentous cells.
   2. **Supplementary tables:** **1**. SWI/SNF and RSC complex in *S. cerevisiae, Human* and *C. albicans.* **2**. List of strains used in this study. **3**. List of primers used in this study. **4**. List of plasmids used in this study.


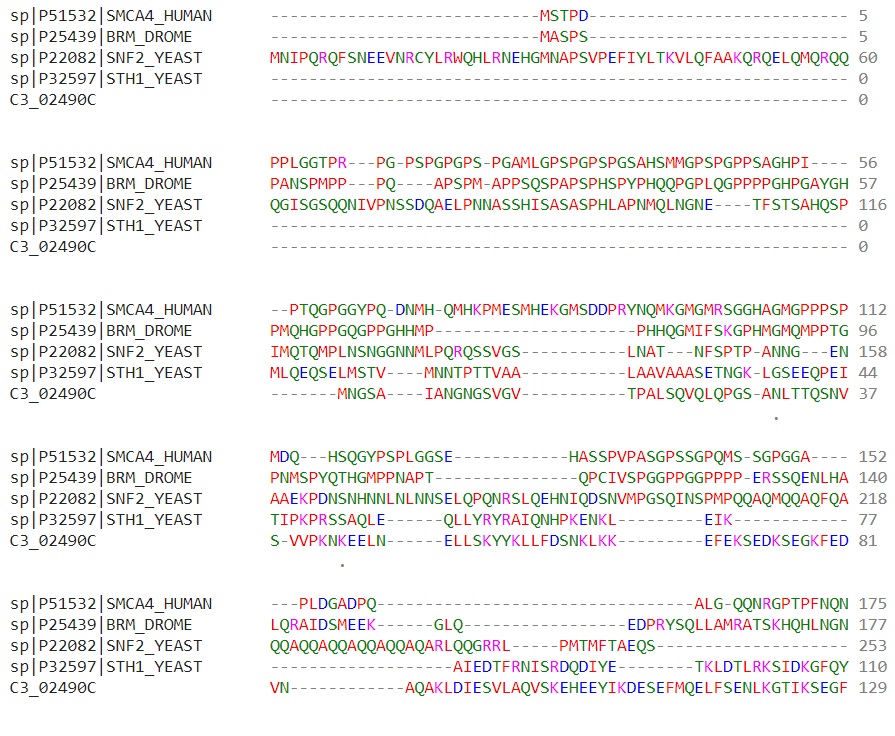

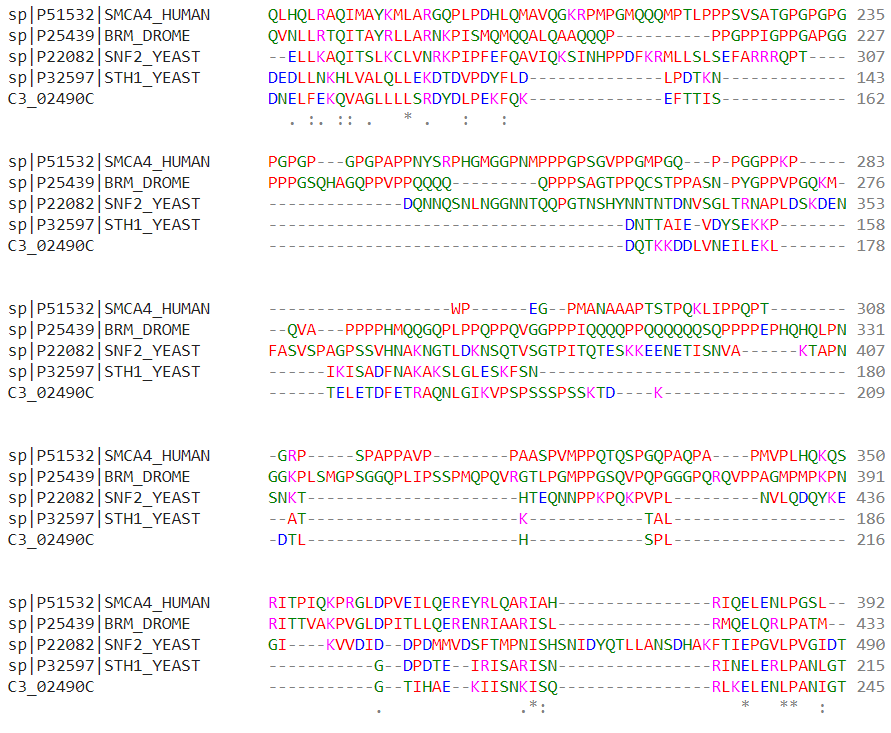


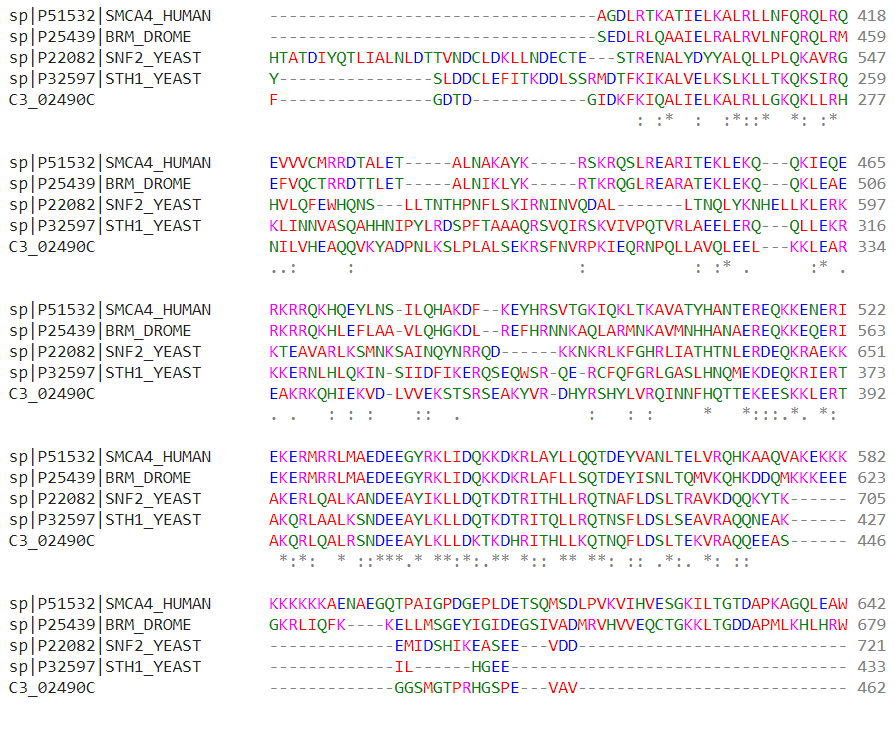

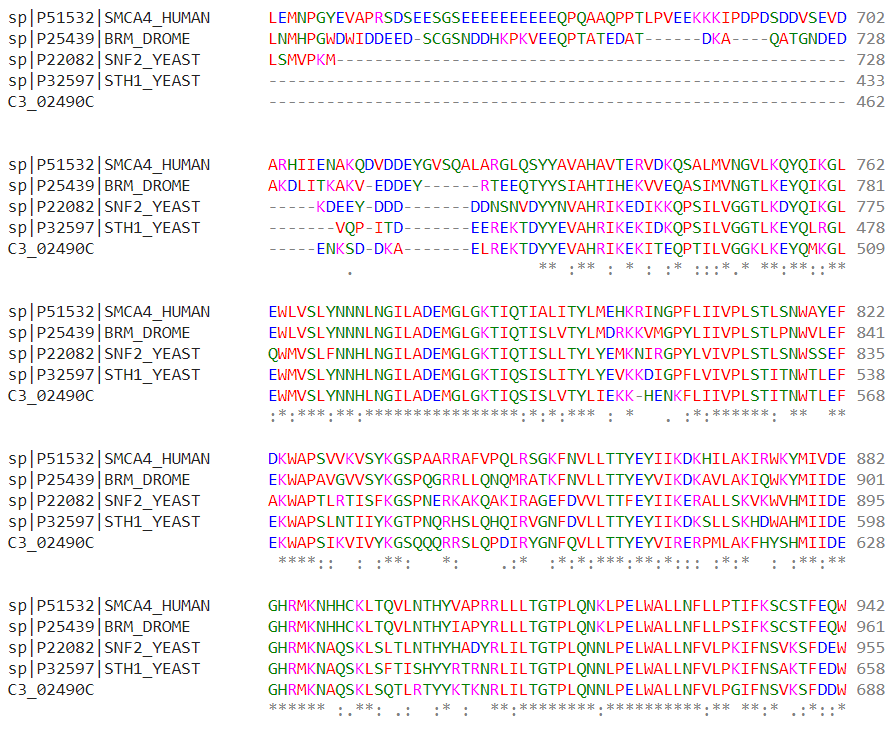

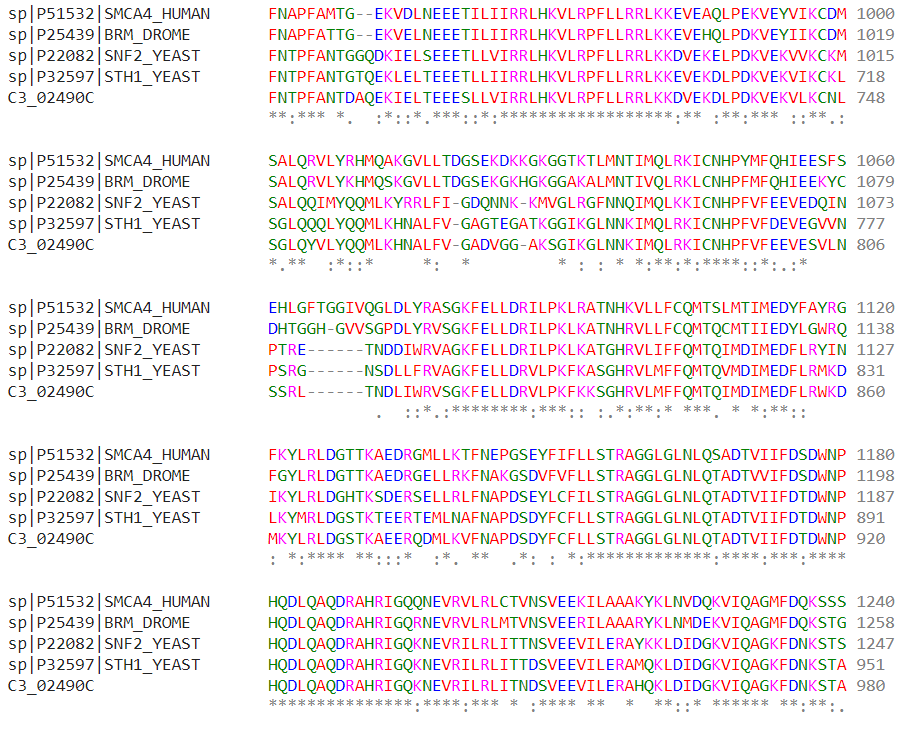

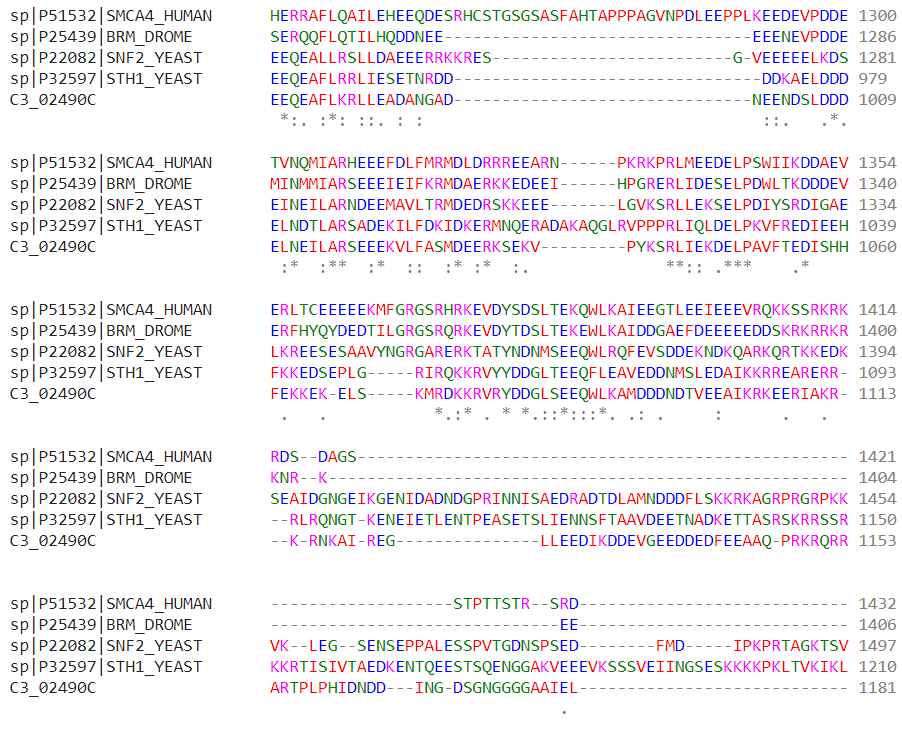

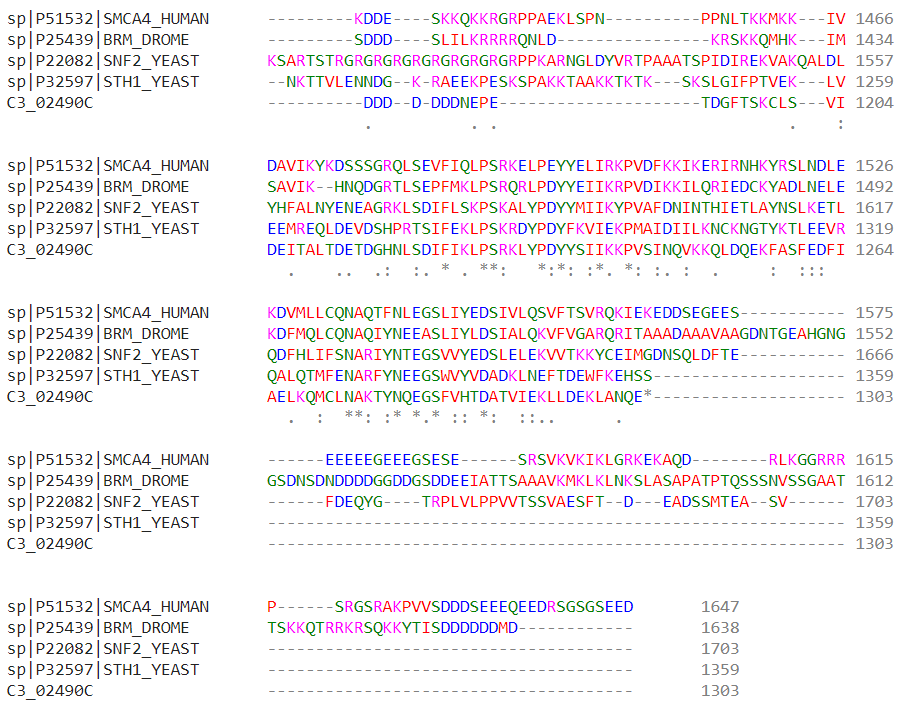


**Figure S1. C3_02490C of *C. albicans* codes for the putative Sth1.** Multiple sequence alignment of Brg1 of *H. sapiens* (SMCA4_HUMAN), Brm of *D. menalogaster* (BRM_DROME), Sth1 and Snf2 of *S. cerevisiae* (SC) and the C3_02490C of *C. albicans* are shown. The first four proteins are mentioned with their corresponding uniprot identifier. Identical amino acids which are consereved among these oragnisms are marked with “ ***** ”.


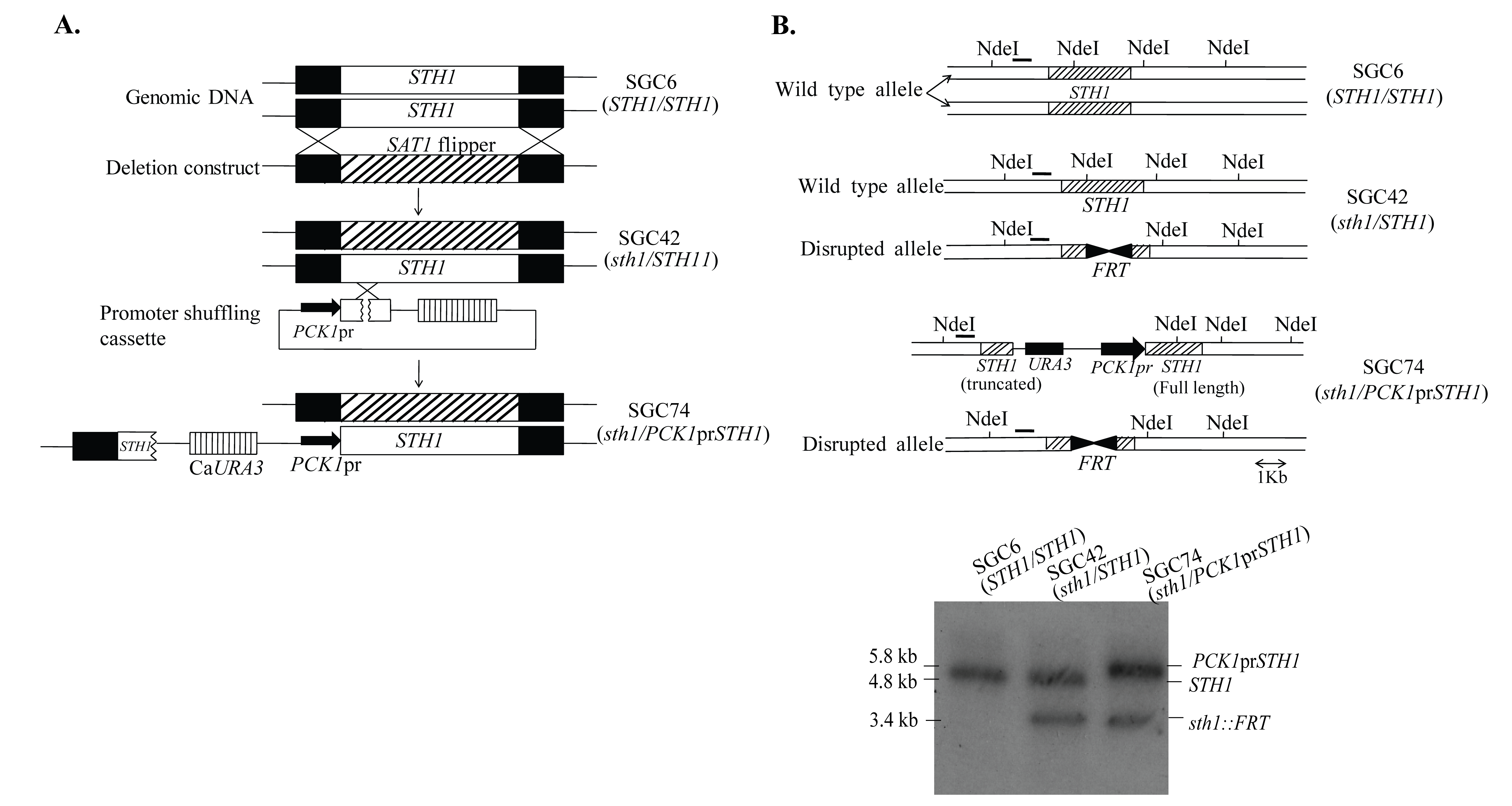


**Figure S2. Construction of conditional mutant of *sth1* where one of the allele of *STH1* is deleted and the other one is placed under the control of *PCK1* promoter. (A)** Schematic of creating the conditional mutant of *sth1*. One of the copies of *STH1* is deleted using the *SAT1* flipper cassette while the native promoter of the second copy is replaced by the promoter of the *PCK1* gene in *C. albicans*. **(B)** Schematic description of Southern blot hybridization to confirm the deletion of *STH1* and shuffling of the native promoter of *STH1* by *PCK1* promoter. Southern blot shows the deletion of the first copy of *STH1* in SGC42 (*sth1/STH1*) and the native promoter of the second copy is shuffled by *PCK1* promoter in SGC74 (*sth1/PCK1*pr*STH1*).


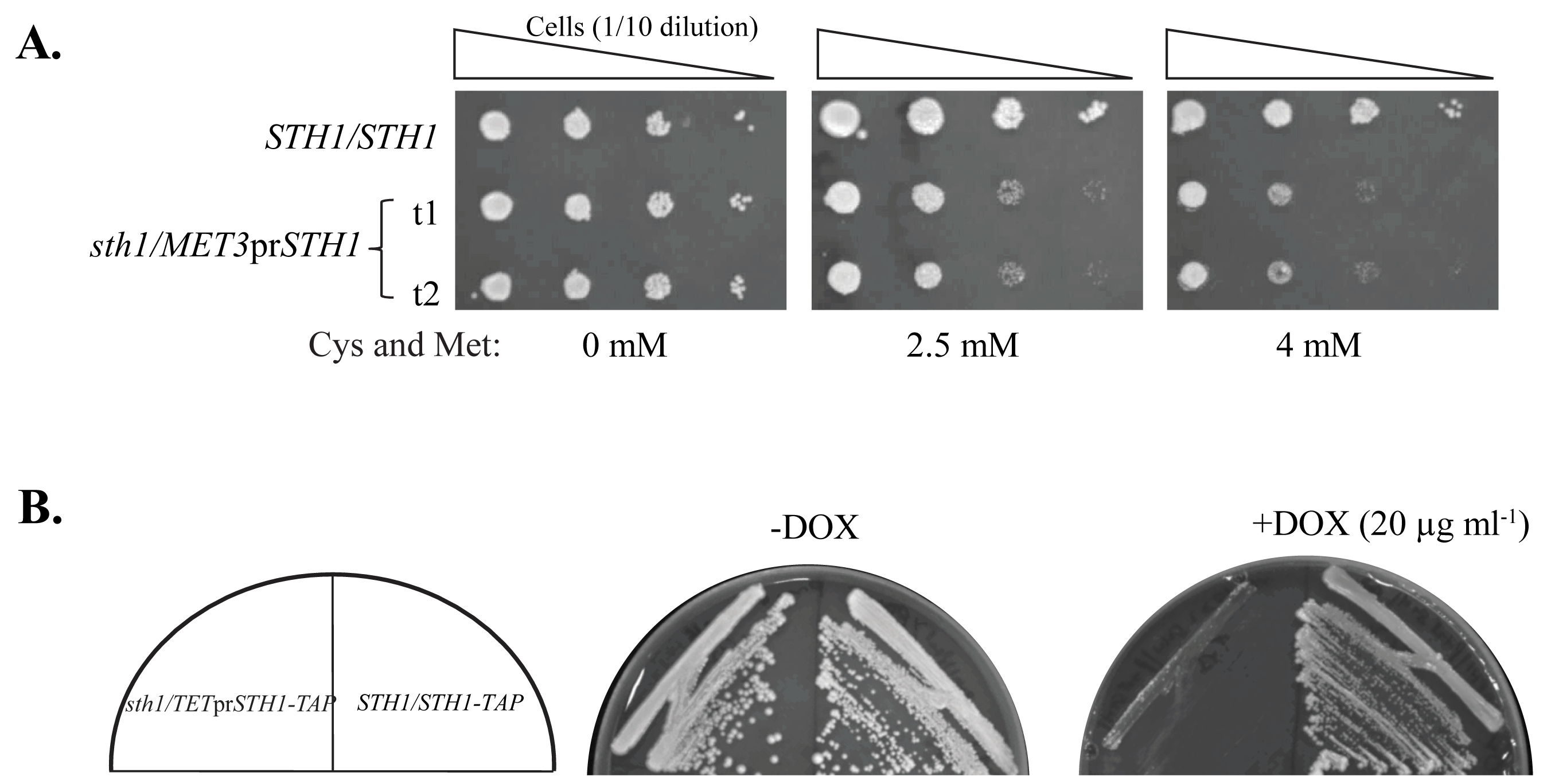


**Figure S3. *STH1* is an essential gene in *C. albicans* shown by repressible *MET3* or *TET* promoter. (A)** The growth of two independent transformants (t1 and t2) of SGC65 (*sth1/MET3*pr*STH1*) are suppressed on 2.5 mM and 4 mM Cysteine (Cys) and Methionine (Met) containing plates, while the wild type SGC6 (*STH1/STH1*) can grow on these plates. **(B)** The strain SGC196 (*sth1/TET*pr*STH1-TAP*) fails to grow in presence of doxycycline (DOX) that shuts off the *TET* promoter.


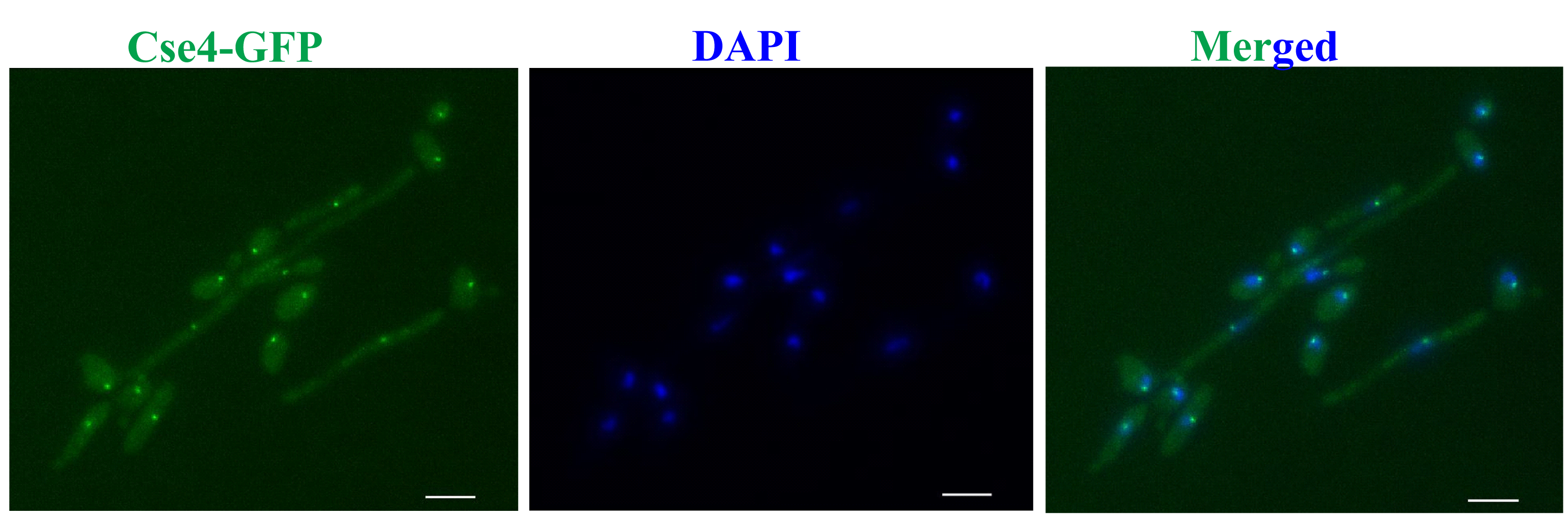


**Figure S4. Cse4-GFP remains clustered in the filamentous cells.** The wild type cells (YJB8675, *STH1/STH1 CSE4/CSE4:GFP:CSE4*) are grown in the spider medium for 4 h before they are visualized under the microscope. The tight knit clustered signals appear in all the cells. Scale bar = 5 µm.

**Table 1: SWI/SNF and RSC complex in *S. cerevisiae, Human* and *C. albicans***

(information retrieved from SGD,CGD, (Monahan et al., 2008))

| ***S. cerevisiae*** | | **Human** | | ***C. albicans*** |
| --- | --- | --- | --- | --- |
| **SWI/SNF** | **RSC** | **BAF** | **PBAF** | **RSC** |
| **Snf2** | **Sth1** | **BRG1 or BRM** | **BRG1** | **C3_02490C** |
| Swi1 |  | BAF250 |  |  |
| Snf5 | Sfh1 | SNF5 | SNF5 | C1_08160W |
| Swi3 | Rsc8 | BAF170, BAF155 | BAF170, BAF155 | C1_14240W |
| Snf12 | Rsc6 | BAF60a | BAF60a or BAF60b |  |
|  |  | BAF53 | BAF53 |  |
| Arp9 | Arp9 |  |  | C3_01030W |
| Arp7 | Arp7 |  |  | C1_03310W |
|  |  | Actin | Actin |  |
| Taf14 |  |  |  |  |
|  | Rsc1 or Rsc2 |  | BAF180 | C1_02750C |
|  | Rsc4 |  |  | C2_00830C |
|  | Rsc9 |  |  | C1_05780W |
|  | Rsc58 |  |  | C2_00570W |
| Swp82 | Rsc7 |  |  |  |
|  |  | BAF57 | BAF57 |  |
| Rtt102 | Rtt102 |  |  |  |
| Snf11 |  |  |  |  |
| Snf6 |  |  |  |  |
|  | Rsc3 |  |  |  |
|  | Rsc30 |  |  |  |
|  | Ldb7 |  |  |  |
|  | Htl1 |  |  |  |

Note: subunits of respective complexes are listed in columns, with orthologous proteins grouped horizontally.

**Table 2: List** of strains used in this study

| Sl. no. | Strain name/id | Parent strain | Genotype | reference |
| --- | --- | --- | --- | --- |
| 1 | SN148 (SGC6) |  | *ura3::imm434/ura3::imm 434 iro1/iro1::imm434 his1::hisG/his1::hisG leu2/leu2* | (Noble and Johnson, 2005) |
| 2 | SGC42 | SN148 | *ura3::imm434/ura3::imm 434 iro1/iro1::imm434 his1::hisG/his1::hisG leu2/leu2, sth1::FRT/STH1* | This study |
| 3 | YJB8675 | BWP17 | *ura3::imm434/ura3::imm 434 iro1/iro1::imm434 his1::hisG/his1::hisG, CSE4/CSE4:GFP:CSE4* | (Joglekar et al., 2008) |
| 4 | J110 | SN148 | *ura3::imm434/ura3::imm 434 iro1/iro1::imm434 his1::hisG/his1::hisG leu2/leu2, mad2::LEU2/mad2::ARG4* | (Thakur and Sanyal, 2011) |
| 5 | SGC51 | 8675 | *ura3::imm434/ura3::imm 434 iro1/iro1::imm434 his1::hisG/his1::hisG leu2/leu2, CSE4/CSE4:GFP:CSE4, sth1::FRT/STH1* | This study |
| 6 | SGC56 | J110 | *ura3::imm434/ura3::imm 434 iro1/iro1::imm434 his1::hisG/his1::hisG leu2/leu2, mad2::LEU2/mad2::ARG4, sth1::FRT/STH1* | This study |
| 7 | SGC65 | SGC42 | *ura3::imm434/ura3::imm 434 iro1/iro1::imm434 his1::hisG/his1::hisG leu2/leu2, sth1::FRT/MET3pr(URA3)-STH1* | This study |
| 8 | SGC66 | SN148 | *ura3::imm434/ura3::imm 434 iro1/iro1::imm434 his1::hisG/his1::hisG leu2/leu2, CSE4:TAP(ARG4)/CSE4* | This study |
| 9 | SGC68 | SGC42 | *ura3::imm434/ura3::imm 434 iro1/iro1::imm434 his1::hisG/his1::hisG leu2/leu2, CSE4-TAP(ARG4)/CSE4,sth1::FRT/STH1* | This study |
| 10 | SGC71 | SGC42 | *ura3::imm434/ura3::imm 434 iro1/iro1::imm434 his1::hisG/his1::hisG leu2/leu2,sth1::FRT/STH1-TAP(ARG4)* | This study |
| 11 | SGC72 | SGC65 | *ura3::imm434/ura3::imm 434 iro1/iro1::imm434 his1::hisG/his1::hisG leu2/leu2,sth1::FRT/MET3pr(URA3)-STH1-TAP(ARG4)* | This study |
| 12 | SGC74 | SGC42 | *ura3::imm434/ura3::imm 434 iro1/iro1::imm434 his1::hisG/his1::hisG leu2/leu2,sth1::FRT/PCK1pr(URA3)-STH1* | This study |
| 13 | SGC78 | SN148 | *ura3::imm434/ura3::imm 434 iro1/iro1::imm434 his1::hisG/his1::hisG leu2/leu2,STH1-TAP(ARG4)/STH1* | This study |
| 14 | SGC79 | SGC74 | *ura3::imm434/ura3::imm 434 iro1/iro1::imm434 his1::hisG/his1::hisG leu2/leu2,sth1::FRT/PCK1pr(URA3)-STH1-TAP* | This study |
| 15 | SGC82 | SGC56 | *ura3::imm434/ura3::imm 434 iro1/iro1::imm434 his1::hisG/his1::hisG leu2/leu2,sth1::FRT/PCK1pr(URA3)-STH1* | This study |
| 16 | SGC88 | SGC51 | *ura3::imm434/ura3::imm 434 iro1/iro1::imm434 his1::hisG/his1::hisG leu2/leu2,sth1::FRT/PCK1pr(URA3)-STH1* | This study |
| 17 | SGC118 | SGC42 | *ura3::imm434/ura3::imm 434 iro1/iro1::imm434 his1::hisG/his1::hisG leu2/leu2,sth1::FRT/STH1,MCD1-TAP(ARG4)/MCD1* | This study |
| 18 | SGC119 | SGC74 | *ura3::imm434/ura3::imm 434 iro1/iro1::imm434 his1::hisG/his1::hisG leu2/leu2,sth1::FRT/PCK1prSTH1,MCD1-TAP(ARG4)/MCD1* | This study |
| 19 | SGC120 | SGC119 | *ura3::imm434/ura3::imm 434 iro1/iro1::imm434 his1::hisG/his1::hisG leu2/leu2,sth1::FRT/PCK1prSTH1-MYC(HIS1),MCD1-TAP(ARG4)/MCD1* | This study |
| 20 | SGC129 | SGC6 | *ura3::imm434/ura3::imm 434 iro1/iro1::imm434 his1::hisG/his1::hisG leu2/leu2 STH1/STH1-MYC(HIS1)* | This study |
| 21 | SGC132 | SN148 | *ura3::imm434/ura3::imm 434 iro1/iro1::imm434 his1::hisG/his1::hisG leu2/leu2,MCD1-TAP(ARG4)/MCD1* | This study |
| 22 | SGC135 | SGC118 | *ura3::imm434/ura3::imm 434 iro1/iro1::imm434 his1::hisG/his1::hisG leu2/leu2,sth1::FRT/STH1-MYC(HIS1),MCD1-TAP(ARG4)/MCD1* | This study |
| 23 | YJB12856 | BWP17 | *ura3::imm434/ura3::imm434 iro1/iro1::imm434 his1::hisG/his1 ::hisG arg4/arg4*  *TUB1/TUB1::GFP-URA3 NOP1/NOP1::RFP-NAT* | (Harrison et al., 2014) |
| 24 | SGC144 | SGC132 | *ura3::imm434/ura3::imm 434 iro1/iro1::imm434 his1::hisG/his1::hisG leu2/leu2,STH1-MYC(HIS1)/STH1,MCD1-TAP(ARG4)/MCD1* | This study |
| 25 | SGC156 | SGC42 | *ura3::imm434/ura3::imm 434 iro1/iro1::imm434 his1::hisG/his1::hisG leu2/leu2,TetR(NAT), sth1::FRT/STH1* | This study |
| 26 | SGC196 | SGC156 | *ura3::imm434/ura3::imm 434 iro1/iro1::imm434 his1::hisG/his1::hisG leu2/leu2, sth1/TETpr(NAT)-STH1-TAP(ARG4)* | This study |
| 27 | SGC214 | YJB12856 | *ura3::imm434/ura3::imm434 iro1/iro1::imm434 his1::hisG/his1 ::hisG arg4/arg4, TUB1/TUB1::GFP-URA3 NOP1/NOP1::RFP-NAT sth1::FRT/STH1* | This study |
| 28 | SGC227 | SGC56 | *ura3::imm434/ura3::imm 434 iro1/iro1::imm434 his1::hisG/his1::hisG leu2/leu2, mad2::LEU2/mad2::ARG4, sth1::FRT/PCK1pr(URA3)-STH1* | This study |
| 29 | SGC234 | SGC214 | *ura3::imm434/ura3::imm434 iro1/iro1::imm434 his1::hisG/his1 ::hisG arg4/arg4 TUB1/TUB1::GFP-URA3 NOP1/NOP1::RFP-NAT sth1::FRT/PCK1pr(ARG4)-STH1* | This study |
| 30 | SGC258 | SGC68 | *ura3::imm434/ura3::imm 434 iro1/iro1::imm434 his1::hisG/his1::hisG leu2/leu2, CSE4-TAP(ARG4)/CSE4, sth1::FRT/PCK1pr(ARG4)-STH1* | This study |
| 31 | SGC260 | SGC66 | *ura3::imm434/ura3::imm 434 iro1/iro1::imm434 his1::hisG/his1::hisG leu2/leu2, CSE4:TAP(ARG4)/CSE4,STH1-MYC(HIS1)/STH1* | This study |
| 32 | SGC261 | SGC68 | *ura3::imm434/ura3::imm 434 iro1/iro1::imm434 his1::hisG/his1::hisG leu2/leu2, CSE4-TAP(ARG4)/CSE4,sth1::FRT/STH1-MYC(HIS1)* | This study |
| 33 | SGC262 | SGC258 | *ura3::imm434/ura3::imm 434 iro1/iro1::imm434 his1::hisG/his1::hisG leu2/leu2, CSE4-TAP(ARG4)/CSE4, sth1::FRT /PCK1pr(ARG4)-STH1-MYC(HIS1)* | This study |

**Table 3: List** of primers used in this study

| Sl. no. | Primer name | Sequence | Description |
| --- | --- | --- | --- |
| 1 | P14 | GAGAAACTGCAGCGGATAACATATTCATACGTGG | 1.8 kb DS of ATG of *STH1* ORF-RP |
| 2 | P18 | GAGACGGGGTACCCGCTCACACTATTTGCGTGT | FP to amplify US sequence of *STH1* for cloning using KpnI |
| 3 | P19 | GAGACCGCTCGAGCTGACCCATTCATAATGATC | RP to amplify US sequence of *STH1* for cloning using XhoI |
| 4 | P20 | GAGAAAGCGGCCGCGTGGGGAAATACAAGAATCG | FP to amplify DS sequence of *STH1* for cloning using NotI |
| 5 | P21 | GAGAGGCGAGCTCGCAATTCTAGATAACCTCCC | RP to amplify DS sequence of *STH1* for cloning using SacI |
| 6 | P22 | CACGCTAGACAAATTCTTCC | 253 bp from the start of *SAT1* flipper cassette-FP |
| 7 | P23 | CAGCTGATACCATAGTTTGA | 940 bp US of ATG of *STH1* ORF-FP |
| 8 | P41 | GTGTCTTAATGCAAAGACTTATAATCAAGAAGGGTCATTTGTACATACTGATGCTACTGTTATTGAGAAATTACTTGATGAGAAATTAGCTAATCAAGAAGGTCGACGGATCCCCGGGTT | FP to amplify *STH1-TAP-ARG4* or *STH1-MYC-HIS1* from pFA-*TAP-ARG4* or pFA-*MYC-HIS1* (refer Supplementary Table 4) respectively |
| 9 | P42 | AGTACAACCAAAAAATAAAATAATAATAATAACAAAAGGAGACGACGATTCTTGTATTTCCCCACTAAATTTATTATTATTATTATTATTATTATTATTATCGATGAATTCGAGCTCGTT | RP to amplify *STH1-TAP-ARG4* or *STH1-MYC-HIS1* from pFA-*TAP-ARG4* or pFA-*MYC-HIS1* (refer Supplementary Table 4) respectively |
| 10 | P43 | CATCAAACTTCCATCACGTA | 244 bp US of stop codon of *STH1*-FP to confirm tagging of Sth1 at C terminus |
| 11 | P44 | TGTTGGAGACAATTTGCAAC | 377 bp DS of stop codon of *STH1*-RP to confirm tagging of Sth1 at C terminus |
| 12 | P45 | GACAAGGGAAGCAATCTTAA | 179 bp upstream of *PCK1* gene-FP to confirm the shuffling of promoter by *PCK1* promoter |
| 13 | P52 | CTAGTGCAAGACCCTCATAGAAGC | FP from *CEN7* for ChIP experiment  Coordinates:  427538-427561 |
| 14 | P53 | CCTGACACTGTCGTTTCCCATAGC | RP from *CEN7* for ChIP experiment  Coordinates: 427361-427384 |
| 15 | P54 | ACTCGCCTTCCCCTCCTTTAAATAG | FP from 126 Mb away from *CEN7* for ChIP experiment (FP of NC7)  Coordinates: 299419  - 299443 |
| 16 | P55 | CCACTACTACGACTGTGGATTCACT | RP from 126 Mb away from *CEN7* for ChIP experiment (RP of NC7)  Coordinates: 299510-299534- |
| 17 | P62 | AACTAATAATTGCATTTCTTTAGGTCAAATCCCACAAGAAACAACAATTGCTGGAGATATCAGCATCACTTCAAGAGACAGATTATTTAGTCAGTTTGTAGGTCGACGGATCCCCGGGTT | FP to amplify *MCD1-TAP-ARG4* cassette from pFA-*TAP-ARG4* (refer Supplementary Table4) |
| 18 | P63 | ACTGTTTACTCCATTGATGATTCAAAGTTTTCCAGATTTGAAAAAGATGATTCCCATTTTGATAAAGGTATATAACTAGTGTACATTTAATATTATTCTATCGATGAATTCGAGCTCGTT | RP to amplify *MCD1-TAP-ARG4* cassette from pFA-*TAP-ARG4* (refer Supplementary Table 4) |
| 19 | P64 | CAAGGATCGCATCAATTCTA | 166 bp US of stop codon of *MCD1* (*ORF19.7634*)-FP to confirm the tagging of Mcd1 at C terminus |
| 20 | P65 | TATCGAGTCATTCTGAATGG | 380 bp DS of stop codon of *MCD1* (*ORF19.7634*)-RP to confirm the tagging of Mcd1 at C terminus |
| 21 | P66 | ATCATGCCATTCTTGTCTGA | Primer within *ARG4*-RP |
| 22 | P67 | TCGATGTTGTCGAATTGTTC | Primer within *HIS1*-RP |
| 23 | P93 | TAGTGCTTCAACTACCCCAG | 2424 bp US of *STH1* ORF, FP for amplifying probe for checking *STH1* deletion by southern blot |
| 24 | P94 | CTTGAGCCAACAGAATGGTG | 2112 bp US of *STH1* ORF, RP for amplifying probe for checking *STH1* deletion by southern blot |
| 25 | P96 | CAGAGCAATGGCCCTTGTGATTGT | RP for non-*CEN* probe outside 1 kb of *CEN7*, for MNase experiment, coordinates: 429656-429679 |
| 26 | P97 | CAAGCTGCCTTGTCAGGCAAAGCATC | FP for non-*CEN* probe outside 1 kb of *CEN7*, for MNase experiment, coordinates: 431064-431089 |
| 27 | P98 | CCACCTCTGCACTAATCTACAATGC | FP for *CEN* probe, within *CEN7*, for MNase experiment, coordinates: 426432-426456 |
| 28 | P113 | CCTCAATTACCTTGCAGTAGTC | FP for amplifying NC3 for ChIP experiment, coordinates: 1357847- 1357868 |
| 29 | P114 | CAATCAAGTACAGCGCAAAC | RP for amplifying NC3 for ChIP experiment, coordinates: 1357964-1357983 |
| 30 | P117 | GAGTTTTGTGTAATGACCAGC | FP for amplifying NC4 for ChIP experiment, coordinates: 862633- 862653 |
| 32 | P118 | TCACTTTGTTGTATTTTGTGG | RP for amplifying NC4 for ChIP experiment, coordinates: 862771  - 862791 |
| 33 | P119 | ACGCAATTTCACCAATTATAGTC | FP for amplifying NC6 for ChIP experiment, coordinates: 166381- 166403 |
| 34 | P120 | GGCAGGTTTGTAAGTAAATAATG | RP for amplifying NC6 for ChIP experiment, coordinates: 166481-166503 |
| 35 | SA27 | GGTATTGTTTTGGATTCTGGTG | FP for amplifying *ACT1* for ChIP experiment, coordinates: 3012424-3012445 |
| 36 | SA28 | CAAGTCTCTACCAGCCAAATC | RP for amplifying *ACT1* for ChIP experiment, coordinates: 3012511-3012531 |
| 37 | SA43 | CAAGTCTGGTCGTTTCCG | FP for amplifying *CEN5* for ChIP experiment, coordinates: 469548-469566 |
| 38 | SA44 | GCAACCAATACAGGTTCCA | RP for amplifying *CEN5* for ChIP experiment, coordinates: 469696-469714 |

FP- forward primer, RP- reverse primer, US- upstream, DS- downstream, coordinates of primers for ChIP experiments are taken from assembly 22.

**Table 4: List of plasmids used in this study**

| Sl. no. | Plasmid | Description | Reference |
| --- | --- | --- | --- |
| 1 | pSFS2a | *SAT1* flipper cassette, chloramphenicol selection | (Reuss et al., 2004) |
| 2 | pCaDIS | *MET3* promoter and *URA3* marker, ampicillin selection | (Care et al., 1999) |
| 3 | pCaDIS-*STH1* | 1832 bp of *STH1* starting from ATG cloned in pCaDIS between *Bam*HI and *Pst*I, ampicillin selection | This study |
| 4 | pSFS2a-US_*STH1* | 570 bp US (amplified by P18, P19) of *STH1* ORF cloned into pSFS2a between KpnI and XhoI sites, chloramphenicol selection | This study |
| 5 | pSFS2a-US.DS_*STH1* | 499 bp of DS (amplified by P20, P21) of *STH1* ORF cloned into pSFS2a-US_*STH1* between NotI and SacI, chloramphenicol selection | This study |
| 6 | pFA-*MYC-HIS1* | *MYC-HIS1* tagging cassette, ampicillin selection | (Lavoie et al., 2008) |
| 7 | pFA-*TAP-ARG4* | *TAP-ARG4* tagging cassette, ampicillin selection | (Lavoie et al., 2008) |
| 8 | pBSK.Cd*ARG4* | Cd*ARG4* cloned as KpnI-XhoI fragment into pBluescript plasmid, ampicillin selection | This study |
| 9 | pLC729 | TetR-*HIS1*, ampicillin selection | (Shapiro et al., 2009) |
| 10 | pLC330 | *TET* promoter-NAT, ampicillin selection | (Shapiro et al., 2009) |
| 11 | pLC330p*STH1*-DS | DS of *STH1* promoter cloned into pLC330 between AflII and SacII sites | This study |
| 12 | pLC330p*STH1*-DS-US | US of *STH1* promoter cloned into pLC330p*STH1*-DS between EcoNI and ApaI sites | This study |

**References**

Care, R. S., Trevethick, J., Binley, K. M., and Sudbery, P. E. (1999). The MET3 promoter: a new tool for Candida albicans molecular genetics. *Mol. Microbiol.* 34, 792–8.

Harrison, B. D., Hashemi, J., Bibi, M., Pulver, R., Bavli, D., Nahmias, Y., et al. (2014). A tetraploid intermediate precedes aneuploid formation in yeasts exposed to fluconazole. *PLoS Biol.* 12, e1001815. doi:10.1371/journal.pbio.1001815.

Joglekar, A. P., Bouck, D., Finley, K., Liu, X., Wan, Y., Berman, J., et al. (2008). Molecular architecture of the kinetochore-microtubule attachment site is conserved between point and regional centromeres. *J. Cell Biol.* 181, 587–594. doi:10.1083/jcb.200803027.

Lavoie, H., Sellam, A., Askew, C., Nantel, A., and Whiteway, M. (2008). A toolbox for epitope-tagging and genome-wide location analysis in Candida albicans. *BMC Genomics* 9, 578. doi:10.1186/1471-2164-9-578.

Monahan, B. J., Villén, J., Marguerat, S., Bähler, J., Gygi, S. P., and Winston, F. (2008). Fission yeast SWI/SNF and RSC complexes show compositional and functional differences from budding yeast. *Nat. Struct. Mol. Biol.* 15, 873–80. doi:10.1038/nsmb.1452.

Noble, S. M., and Johnson, A. D. (2005). Strains and Strategies for Large-Scale Gene Deletion Studies of the Diploid Human Fungal Pathogen Candida albicans. 4, 298–309. doi:10.1128/EC.4.2.298.

Reuss, O., Vik, A., Kolter, R., and Morschhäuser, J. (2004). The SAT1 flipper, an optimized tool for gene disruption in Candida albicans. *Gene* 341, 119–27. doi:10.1016/j.gene.2004.06.021.

Shapiro, R. S., Uppuluri, P., Zaas, A. K., Collins, C., Senn, H., Perfect, J. R., et al. (2009). Hsp90 Orchestrates Temperature-Dependent Candida albicans Morphogenesis via Ras1-PKA Signaling. *Curr. Biol.* 19, 621–629. doi:10.1016/j.cub.2009.03.017.

Thakur, J., and Sanyal, K. (2011). The Essentiality of the Fungus-Specific Dam1 Complex Is Correlated with a One-Kinetochore-One-Microtubule Interaction Present throughout the cell cycle, Independent of the Nature of a Centromere. *Eukaryot. Cell* 10, 1295–1305. doi:10.1128/EC.05093-11.
